# Comparison of Multiple Displacement Amplification (MDA) and Multiple Annealing and Looping-Based Amplification Cycles (MALBAC) in Single-Cell Sequencing

**DOI:** 10.1371/journal.pone.0114520

**Published:** 2014-12-08

**Authors:** Minfeng Chen, Pengfei Song, Dan Zou, Xuesong Hu, Shancen Zhao, Shengjie Gao, Fei Ling

**Affiliations:** 1 School of Bioscience and Bioengineering, South China University of Technology, Guangzhou, 510006, China; 2 BGI-Shenzhen, Shenzhen, 518083, China; 3 The fourth people's hospital of Shenzhen (Futian hospital), Shenzhen, 518033, China; 4 School of Computer, National University of Defense Technology, Changsha, 410073, China; 5 State Key Laboratory of Agrobiotechnology and School of Life Sciences, The Chinese University of Hong Kong, Hong Kong, China; 6 Department of Molecular Medicine, Aarhus University Hospital, Aarhus, Denmark; Albert Einsten College of Medicine, United States of America

## Abstract

Single-cell sequencing promotes our understanding of the heterogeneity of cellular populations, including the haplotypes and genomic variability among different generation of cells. Whole-genome amplification is crucial to generate sufficient DNA fragments for single-cell sequencing projects. Using sequencing data from single sperms, we quantitatively compare two prevailing amplification methods that extensively applied in single-cell sequencing, multiple displacement amplification (MDA) and multiple annealing and looping-based amplification cycles (MALBAC). Our results show that MALBAC, as a combination of modified MDA and tweaked PCR, has a higher level of uniformity, specificity and reproducibility.

## Introduction

Single-cell studies by whole genome amplification were proposed to investigate cellular behaviors from a broad range of environmental and clinical specimens. With the advance of next generation sequencing technologies, single-cell sequencing is expected to result in a novel understanding of genomic stability at the level of per cell cycle in various cell types. There have been several studies on genome-wide sequencing of single cells, which opened an era for investigatin*g* haplotype [1]–[3] and genomic variability [4], [5], especially in monitoring stem cells and tracking of tumor evolution [6]. The quality and quantity of DNA samples are critical for high-throughput sequencing and genetic analysis. Technically, these studies rely on whole-genome amplification from a single cell to generate enough DNA for sequencing library construction [7]–[11]. However, the experimental methods employed in previous reports are prone to amplification bias in some degree, resulting in non-random genome coverage. Thus, a crucial innovation in single-cell sequencing is to put in place strategies for whole-genome amplification with minimal amplification bias.

Polymerase chain reaction (PCR) based amplification introduces sequence-dependent bias due to the exponential amplification with random primers [7], [8], [12]. Multiple displacement φ29 DNA polymerase with random primers under isothermal conditions has been widely improved and widely applied in single-cell sequencing projects [9], [13], [14]. Even though, some bias that resulted from non-linear amplification and chimera still exists [15], [16]. The polymerase displaces downstream DNA strand to extend the growing strand, which, results in a branching form of amplification [17]. Compared with PCR-based methods, MDA reduces amplification bias by three to four orders of magnitude and generates much longer amplicons with average length >12 kb [13]. However, amplification bias still exists in MDA method, which can confuse the heterozygous loci as homozygous ones. Owing to this reason, multiple single-cell sequencings and bulk DNA extractions become necessary for the application of MDA in detecting single nucleotide polymorphisms in diploid genomes [6], [18].

An alternative method of whole genome amplification, so-called multiple annealing and looping-based amplification cycles (MALBAC), emerged recently [5], [17]. Combining advantage features of MDA and tweaked PCR, MALBAC substantially reduces experimental bias related to non-linear amplification. Amplification with MALBAC is initiated with a batch of random primers that can evenly hybridize to the templates at 0°C. Each random primer has a common 27-nucleotide sequence and 8 variable nucleotides. After a 5-cycle initial reaction, specific DNA polymerases with strand-displacement activity were used to generate semi-amplicons at 65°C. The same primers having complementary ends were then used to generate full amplicons after the annealing at 94°C. As the common sequence of random primer on terminal can form pan-like amplicons, enough quantity of DNA production for sequencing will be obtained after an 18-cycle regular PCR amplification. By this approach, MALBAC method can prevent a lot of random amplification bias. Since MALBAC can evenly amplify all the chromosomes in a diploid species, it improves the accuracy of heterozygous SNP calling.

Although it reported that MALBAC is superior to MDA in cancer cells [5], the efficacy of its application in diverse cell types needs to be evaluated. To provide a comprehensive comparison of the two methods, we analyzed six human sperm samples from recent single-cell sequencing projects, of which three amplified using MDA [3] and three using MALBAC [5], respectively.

## Materials and Methods

### Public data availability

The sequencing reads of single sperm cells employed in our analysis was downloaded from NCBI Short Read Archive (SRA). The accession numbers of MDA and MALBAC samples were SRP013494 and SRP017186, respectively. The whole genome sequencing reads of a blood tissue, which was from the same person with the MALBAC samples, were downloaded with the accession number SRR618666. All samples were sequenced using generated from short insert libraries on Illumina HiSeq 2000 with an average of 26.7 Gb and a standard deviation of 1.38 Gb according to SRA record. The sample information was retrieved from the corresponding instruction in NCBI (**S1 Table**).

### Analysis of *K*-mer frequency

The uniformity and reproducibility among different samples can be reflected by the distribution of *K*-mer frequency. Disk-based *K*-mer counting method [19] was utilized to analyze *K*-mer frequency in the sequencing data of each sample. *K* was set to 25 in our analysis. Theoretic value of λ in Poisson distribution was calculated as: 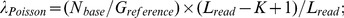



Here: N_base_ represents the total base number; L_read_ represents the read length, *K* is 25 as mentioned above, and G_reference_ denotes the reference genome size 3095.7 Mb for GRCh37. *K*-mer coverage depth was determined as described by Li *et al.* [20].

### Reads alignment

The alignment software BWA v0.6.1 [21] was used to align short reads of each sample to the human reference GRCh37. The quality trimming parameter of BWA was set to 10. A pair of reads were used in subsequent analysis only if one or both reads in the pair was uniquely mapped to the reference.

### Calculating genome coverage

The uniquely mapped reads described above were used to calculate the genome coverage for each sample. A sliding window of 1 Mb was designed to count uniquely mapped reads as it slipped along the reference. The sequencing coverage and depth of each window was calculated using SAMTOOLS [22]. Kendall's test coefficient τ was used to measure the difference of sequencing coverage and depth on different autosomes.

### SNP calling

The maximum likelihood estimation method was applied to population SNP calling. Genotype likelihood of each genomic site for each line was calculated by SAMTOOLS and BCFTOOLS [22] for each sample. The SNP filtering parameters for BCFTOOLs are “varFilter -Q 50 -d 5 -D 60”, which mean that the minimum mapping quality, the minimum and maximum depth for each SNP is 50, 5 and 60, respectively. The NCBI Human dbSNP (Build 142) [23] was used to estimate the quality of SNP calling.

## Results

### The distribution of *K*-mer frequency

**Figure 1:**
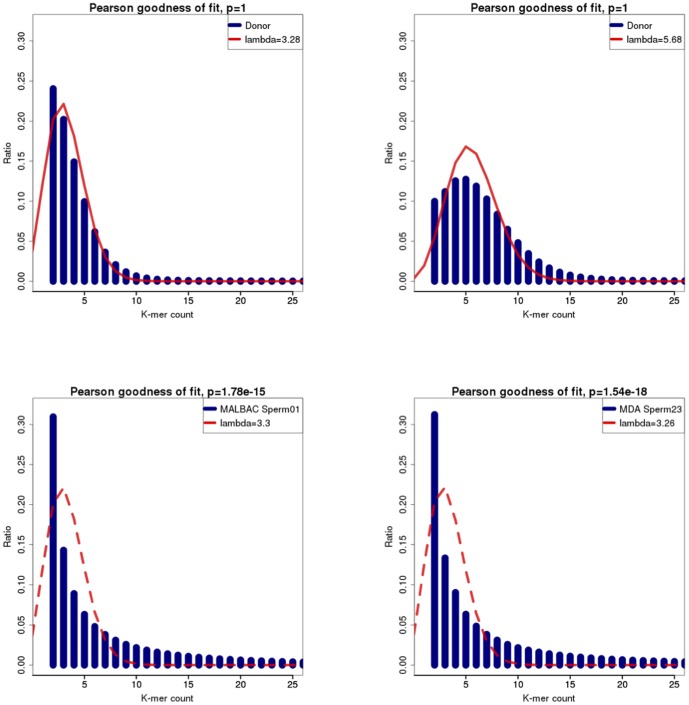
Histogram shows the accordance of *K*-mer frequency with theoretical Poisson.

Approximately, 27 Gb and 26 Gb raw sequencing data was downloaded for each MDA-based and MALBAC-based sample, respectively (**S1 Table**). We also retrieved 46 Gb whole-genome sequencing reads from blood tissues for our analysis. The goodness of fit between the theoretical and practical distribution of *K*-mer frequency reflects the sequencing uniformity and reproducibility [24]. Histogram figures of practical and theoretical Poisson distribution of *K*-mer frequency tell a better consistency in whole-genome sequencing blood sample than the sperms (**Fig. 1**). Only *K*-mer frequency from blood sample matches theoretical Poisson distribution very well, indicating whole-genome amplification methods still need technical improvements (**S2 Table**).

**Figure 2:**
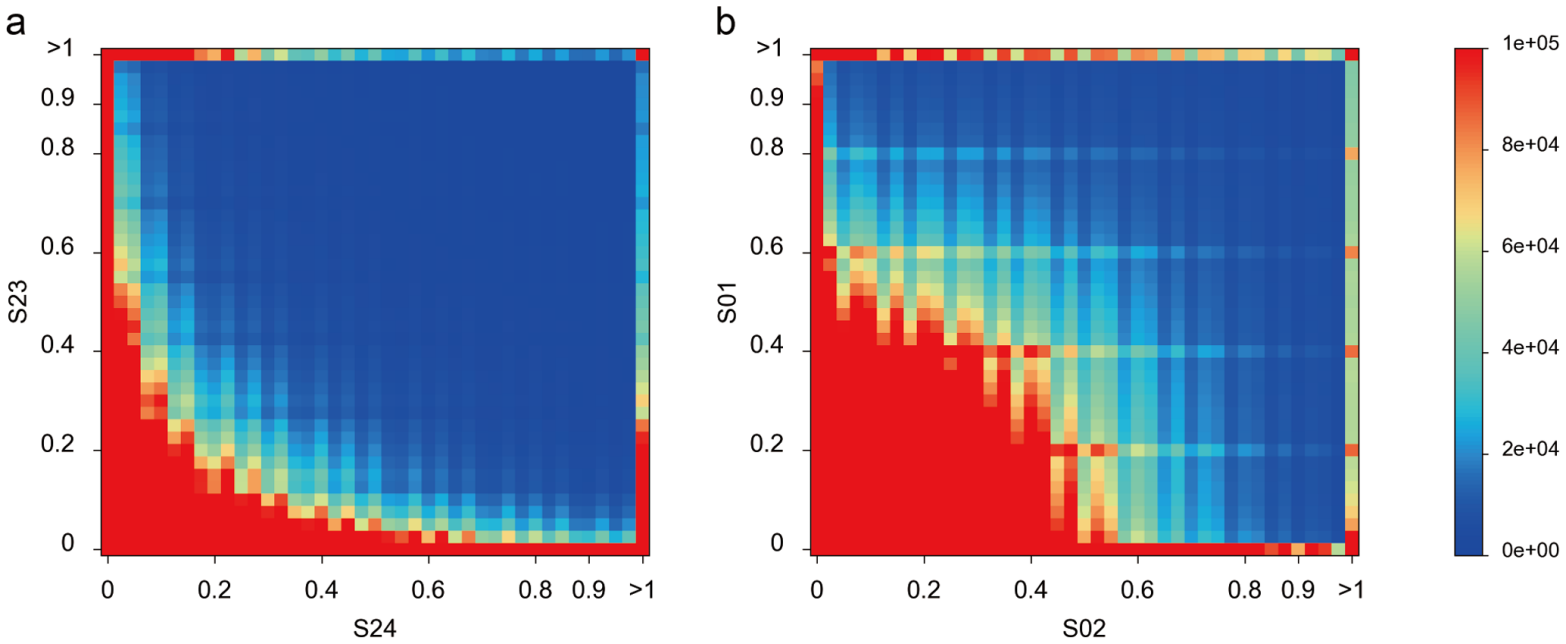
The joint distribution of *K*-mer frequency in two randomly paired samples.

However, the Poisson distribution could be biased due to reasons such as genome composition, sequencing methods, assembly errors, *etc*. To access the two methods without Poisson assumption, we chose the non-parametric Kolmogorov-Smirnov (K-S) test to compare two samples. The two-sample K-S test is one of the most useful and general nonparametric methods, as it is sensitive to differences in both location and shape of the empirical cumulative distributions of two samples. The *P* values in MDA samples are slightly higher than those in MALBAC samples, revealing a little better randomness in MDA sequencing (**S2 Table**). To further compare the reproducibility of two methods, we assessed the joint distribution of *K*-mer frequency in two randomly paired samples. The theoretical simulation of the joint distribution shaped a quadrant with a radius of 0.5 and center at (0,0). Referring to the main diagonal line, MALBAC sequencing data show a better mirror symmetry than the MAD-based approach (**Fig. 2** **and Figures in** **S1 File**). The quadrant area is almost filled up by the joint distribution of *K*-mer frequency in the MALBAC-based sequencing data. Consistent with the results of goodness-of-fit test, MALBAC works better than MDA in amplification uniformity for single-cell sequencing.

### Uniformity of genomic coverage

Short reads of different samples were aligned to the human reference using BWA [15]. Approximately an average of 22 Gb raw data for each sample was successfully mapped to the genome (**Table 1** **and** **S3 Table**). The uniquely mapped reads were retained for subsequent analysis, corresponding to 8.1-fold depth for MDA sequencing sample, 6.9-fold depth for MALBAC, and 12.7-fold depth for blood sample, respectively. Since the blood sample was whole genome sequenced, it can be used as control to evaluate the statistics of genome coverage for MDA and MALBAC based sequencing.

**Table 1:** Global Statistics of single-cell sequencing and mapping in diferent samples.

Sample	Raw Reads	Read length	Mapped reads	Mapped Bases (Mb)	Uniquely mapped reads	Uniquely mapped Bases (Mb)
**MDA 23**	282,171,418	99	258,396,544	24,565	250,520,102	23,816
**MDA 24**	276,998,928	99	243,689,828	23,241	235,286,976	22,436
**MDA 28**	273,358,124	99	250,026,856	23,699	242,764,595	23,013
**Donor**	462,571,116	100	415,071,212	37,802	396,751,691	36,268
**MALBAC 01**	268,487,462	100	254,489,098	21,179	245,501,985	20,470
**MALBAC 02**	267,348,940	100	253,988,896	21,193	245,642,239	20,540
**MALBAC 03**	239,882,962	100	227,489,214	19,165	219,683,435	18,538

Although MALBAC sequencing data were slightly less, their genome coverage is higher than those based on MDA. It is noticed that the mapped reads of MALBAC-based sequencing are short, because of a 27-bp common primer (GTG AGT GAT GGT TGA GGT AGT GTG GAG) were used in the library and then removed after sequencing.

We analyzed the uniformity of genomic coverage between the samples using pairwise Kendall's test. The base coverage is significantly different between MDA and MALBAC sperm samples (*P*<0.01, **S4 Table**). The coefficients τ of genomic coverage among samples with MALBAC are around 0.75, which are considerably higher than those with MDA (∼0.3), indicating a better reproducibility using MALBAC method (**Table 2** **and** **S5 Table**).

**Table 2:** Pairwise Kendall's τ coefficient test of reads coverage of different samples on autosomes.

	MDA 23	MDA 24	MDA 28	Donor	MALBAC 01	MALBAC 02	MALBAC 03
**MDA 23**	1	0.3014	0.3082	0.3029	0.0223**	0.0469	−0.0256*
**MDA 24**		1	0.3067	0.3055	0.0294*	0.0591	−0.0129**
**MDA 28**			1	0.3144	0.0229**	0.0585	−0.0206**
**Donor**				1	0.1904	0.2330	0.1410
**MALBAC 01**					1	0.7754	0.7156
**MALBAC 02**						1	0.7934
**MALBAC 03**							1

* and ** means p more than 1% and 5%. Alternative hypothesis: true τ is not equal to 0.

We also assessed the correlation of sequencing blood with MALBAC and MDA sperm samples, respectively. The coefficients τ of genomic coverage between blood and MALBAC sperms are around 0.15, whereas τ between blood and MDA sperms are around 0.25. The larger coefficients represent that MDA is slightly more random than MALBAC. However, genomic coverage of each MDA sample is lower than that of each MALBAC sample (**S1 Figure**). A higher coverage in MALBAC samples was also observed from the distribution of covered and uncovered genomic regions using *t*-test (**Table 3** **and Figures in** **S2 File**).

**Table 3:** Pairwise Kendall's τ coefficient test of uncovered ratio in 1 M windows of different samples on autosomes.

	MDA 23	MDA 24	MDA 28	Donor	MALBAC 01	MALBAC 02	MALBAC 03
**MDA 23**	1	0.4298	0.4682	0.3867	0.1954	0.2195	0.0906
**MDA 24**		1	0.4709	0.3835	0.2058	0.2336	0.1171
**MDA 28**			1	0.4085	0.1973	0.2245	0.0955
**Donor**				1	0.2503	0.2697	0.1668
**MALBAC 01**					1	0.5241	0.5808
**MALBAC 02**						1	0.5568
**MALBAC 03**							1

**Figure 3:**
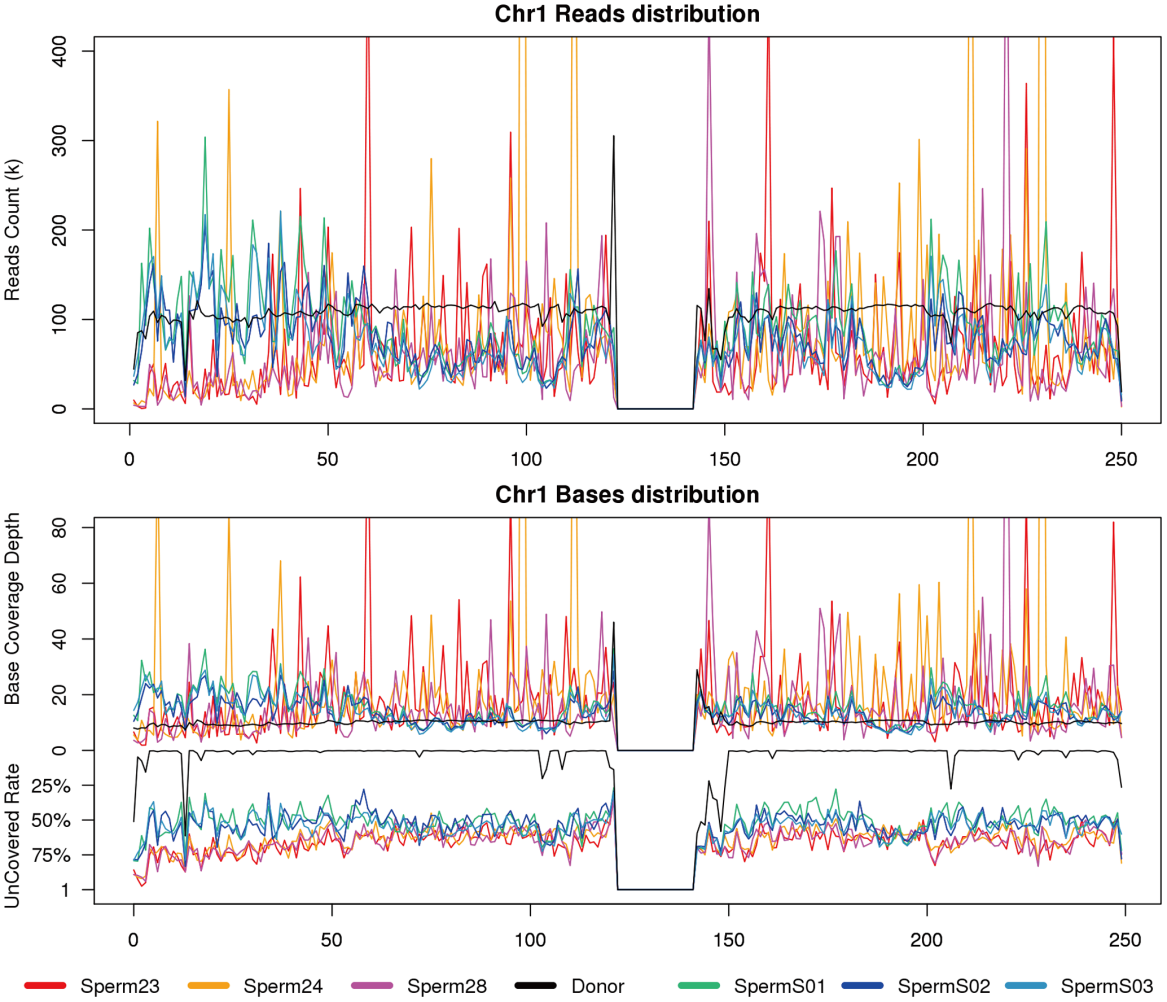
Genomic coverage on chromosome 1 (Chr01). Tilling window size is 1 M. (a) Reads counts in each window; (b) Base coverage depth (upper) and uncovered base rate (lower) in each window. Sperm 23∼28 are MDA samples and Sperm S01∼S03 are MALBAC samples. Refer to supplemental figures for other autosomes.

Although the MALBAC samples showed a higher level of uniformity than MDA samples, regional amplification bias still exists compared with the blood of whole-genome sequencing. Short reads from MDA samples show higher level of random distribution along the chromosomes, indicating lower amplification specificity than MALBAC samples (**Fig. 3a**). To separately survey the covered and uncovered regions, we further compared coverage depth in each sliding window and observed a higher coverage rate in MALBAC samples than other samples in most sliding windows. Examining the average depth in windows, MDA samples have more random peaks, which indicate lower reproducibility than MALBAC samples (**Fig. 3b**).

### Application in SNP identification

**Figure 4:**
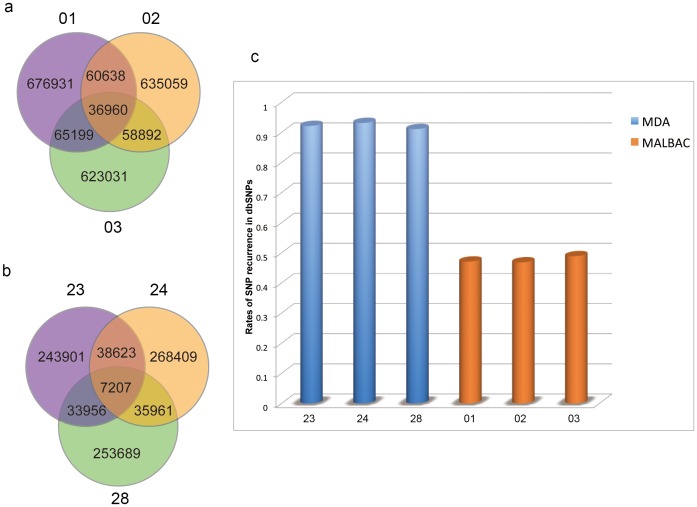
Evaluation of the SNP quality. Venn diagram shows the SNPs share among (a) MALBAC samples and (b) MDA samples; (c) Histogram represents the rates of SNP recurrence in dbSNP.

To evaluate the performance of MALBAC and MDA methods in variation detection, we identified SNPs with the sequencing samples using maximum likelihood estimation. After strict filtering, more high-quality SNPs were detected and shared in MALBAC samples than MDA samples (**Fig. 4a, 4b**). It supported the analysis that MALBAC method gets a better performance in amplification uniformity and reproducibility. Besides, the ratio of heterozygous alleles in MDA samples in lower than those in MALBAC samples in SNP calling (**S6 Table**).

In genetics, mutations can be transitions between purines (A↔G) or pyrimidines (C↔T), or transversions of a purine for a pyrimidine or vice versa. The occurrences of both types in the samples are in line, and there are twice as many possible transitions as transversions (**S2 Figure** **and** **S7 Table**). Oxidative deamination and tautomerization can cause transitions, whereas ionizing radiation and alkylating agents can cause transversions. Because transversions dramatically change their chemical structure, the consequences are often more drastic than those of transitions. Thus, precise acquisition of mutation types is important in the application of amplification methods, especially in cancer researches [25].

The SNP calling quality based on the two amplification methods is another critical concern, in particular for applications in population studies. The quality of SNPs derived from MALBAC and MDA samples were evaluated using NCBI Human dbSNP. It is evident that percentages of SNPs from MALBAC samples validated in dbSNP were significantly higher than those from MDA. As dbSNP accepts submissions of common as well as polymorphic variations, and contains both germline and somatic variations [23], our comparison indicated that the SNP quality of MALBAC approach would be higher than that of MDA.

## Discussion

Whole-genome amplification strategy is imperative for a single-cell sequencing project. We compared the MDA and MALBAC methods from the perspective of uniformity, reproducibility and specificity. Generally, MALBAC offers a better genomic coverage with less amplification bias, as it is a technic improvement based on MDA methods. Using two enzymes strand displacements and PCR amplification, MALBAC probably introduces sequence specific bias, which was evaluated by genomic coverage and sequencing depth in sliding windows. Error rates introduced by *Taq* polymerase (5E-6) in regular PCR in MALBAC will be 10 times higher than that in MDA, considering two round of so-called quansi-linear amplification.

However, MALBAC exhibits a high level of specificity and reproducibility, which facilitate its application in population re-sequencing projects. As genomic regions amplified using MDA show substantial difference among samples, it is hard to apply in genetic map construction based on single sperm sequencing. Instead, MDA will provide more genomic coverage for sequencing if mix more cells.

## Supporting Information

S1 Figure The distribution of sequencing depth for each sample.
(TIF)

S2 Figure Spectrum distribution of allele types in each sample.
(TIF)

S1 Table Sample list with accession numbers.
(DOCX)

S2 Table Goodness-of-fit test to match *K*-mer distribution to Possion probability mass function of the theoretic lambda and compare MDA & MALBAC *K*-mer distribution to the blood *K*-mer distribution with Kolmogorov-Smirnov test.
(DOCX)

S3 Table The ratio of single-cell sequencing and mapping in diferent samples.
(DOCX)

S4 Table Coefficient of variation of coverage in 1Mb windows on autosomes.
(DOCX)

S5 Table Pairwise Kendall's τ coefficient test of base coverage of different samples on autosomes.
(DOCX)

S6 Table Statistics of SNP calling in different samples.
(DOCX)

S7 Table Distribution of allele types in different samples.
(DOCX)

S1 File The joint distribution of *K*-mer frequency in the rest randomly paired samples.
(ZIP)

S2 File Genomic coverage on the rest chromosomes. Tilling window size is 1 M. (a) Reads counts in each window. (b) Base coverage depth (upper) and uncovered base rate (lower) in each window. Sperm 23∼28 are MDA samples and Sperm S01∼S03 are MALBAC samples.
(ZIP)
