# Correction: Comparison of Multiple Displacement Amplification (MDA) and Multiple Annealing and Looping-Based Amplification Cycles (MALBAC) in Single-Cell Sequencing

**DOI:** 10.1371/journal.pone.0124990

**Published:** 2015-04-13

**Authors:** Minfeng Chen, Pengfei Song, Dan Zou, Xuesong Hu, Shancen Zhao, Shengjie Gao, Fei Ling

There are errors in the author affiliations. The affiliations should appear as shown here:

Minfeng Chen^1,2^, Pengfei Song^3^, Dan Zou^4^, Xuesong Hu^2^, Shancen Zhao^2,5^, Shengjie Gao^2,6^, Fei Ling^1^

1 School of Bioscience and Bioengineering, South China University of Technology, Guangzhou, 510006, China, 2 BGI-Shenzhen, Shenzhen, 518083, China, 3 The fourth people’s hospital of Shenzhen (Futian hospital), Shenzhen, 518033, China, 4 School of Computer, National University of Defense Technology, Changsha, 410073, China, 5 State Key Laboratory of Agrobiotechnology and School of Life Sciences, The Chinese University of Hong Kong, Hong Kong, China, 6 Department of Molecular Medicine, Aarhus University Hospital, Aarhus, Denmark
