# Supplementary figures and images for: Comparison of Multiple Displacement Amplification (MDA) and Multiple Annealing and Looping-Based Amplification Cycles (MALBAC) in Single-Cell Sequencing
Source: PLoS One. 2014 Dec 8;9(12):e114520. doi: 10.1371/journal.pone.0114520 (PMC4259343; doi:10.1371/journal.pone.0114520)

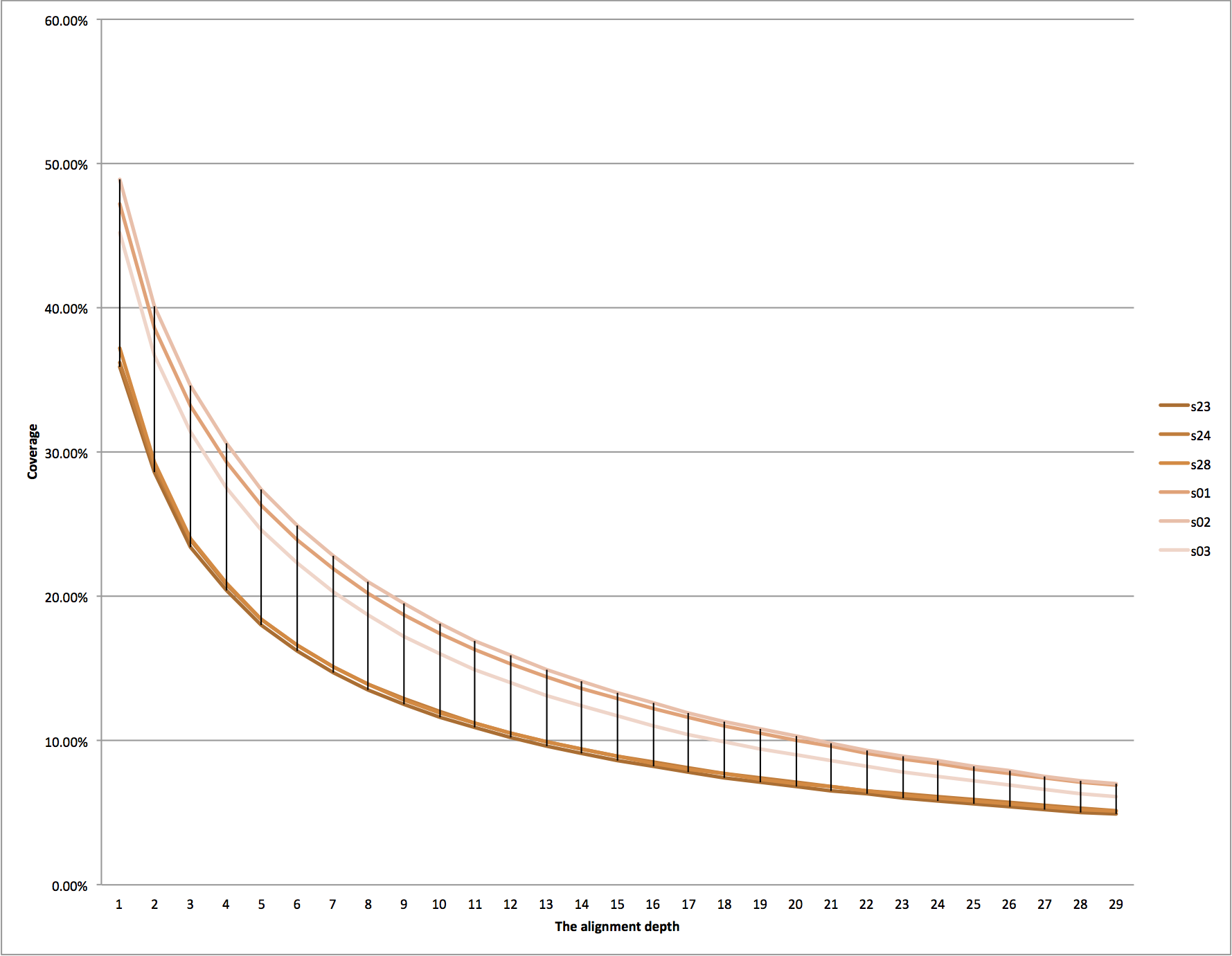

Supplement: S1 Figure — The distribution of sequencing depth for each sample. (TIF) [file pone.0114520.s001.tif]

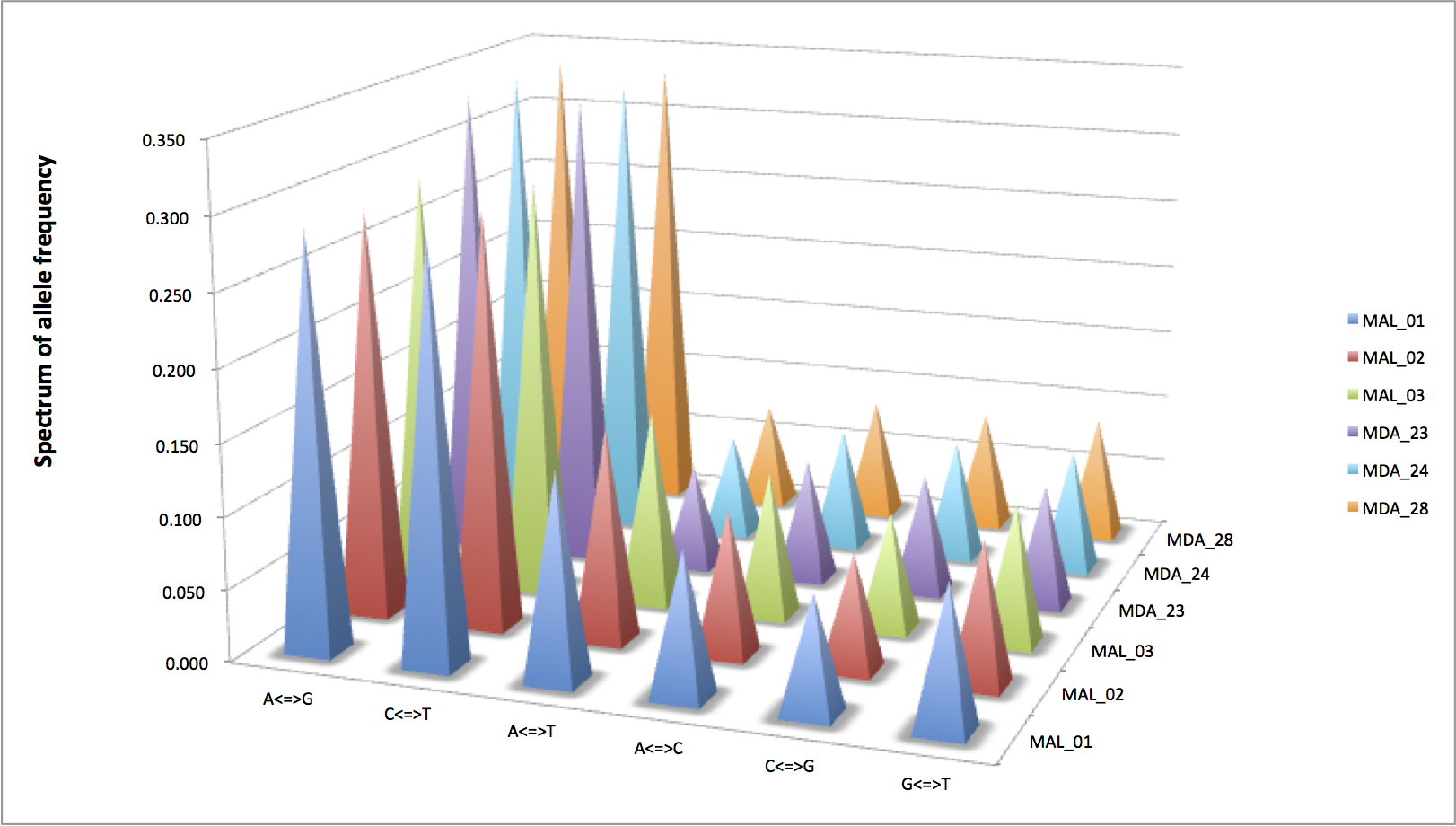

Supplement: S2 Figure — Spectrum distribution of allele types in each sample. (TIF) [file pone.0114520.s002.tif]

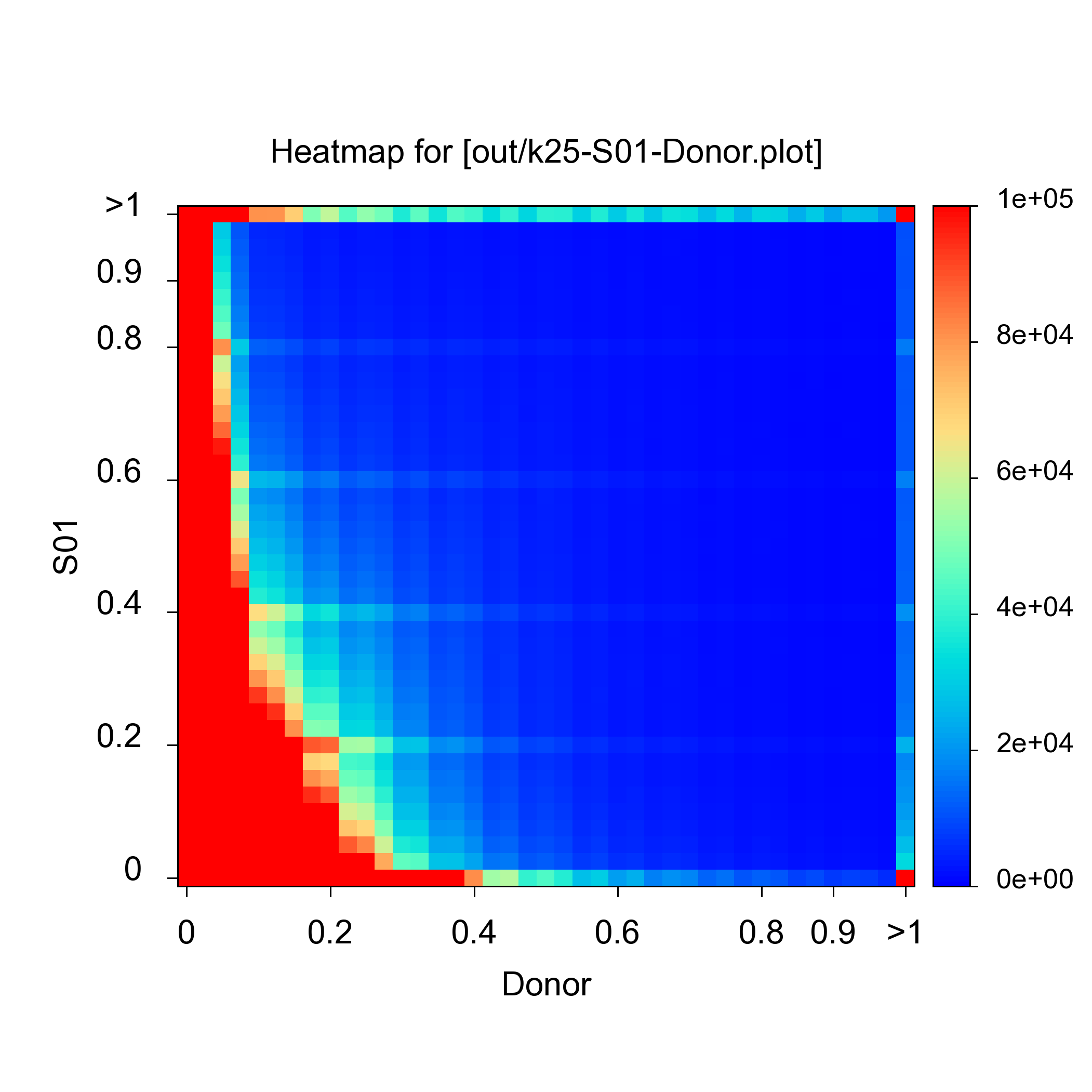

Supplement: S1 File — The joint distribution of K-mer frequency in the rest randomly paired samples. (ZIP) [file pone.0114520.s010.zip › Figure-S1/k25-S01-Donor.plot.png]

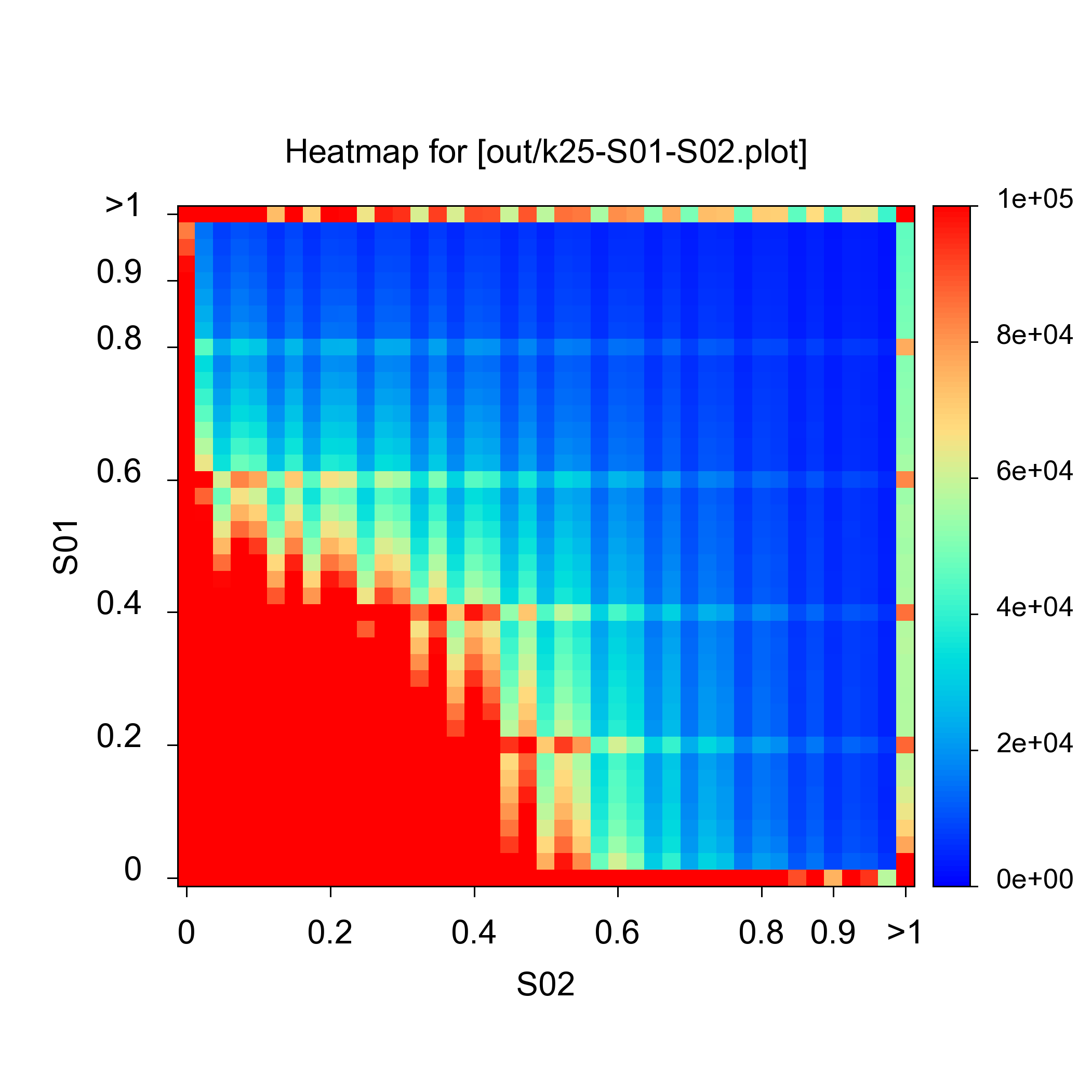

Supplement: S1 File — The joint distribution of K-mer frequency in the rest randomly paired samples. (ZIP) [file pone.0114520.s010.zip › Figure-S1/k25-S01-S02.plot.png]

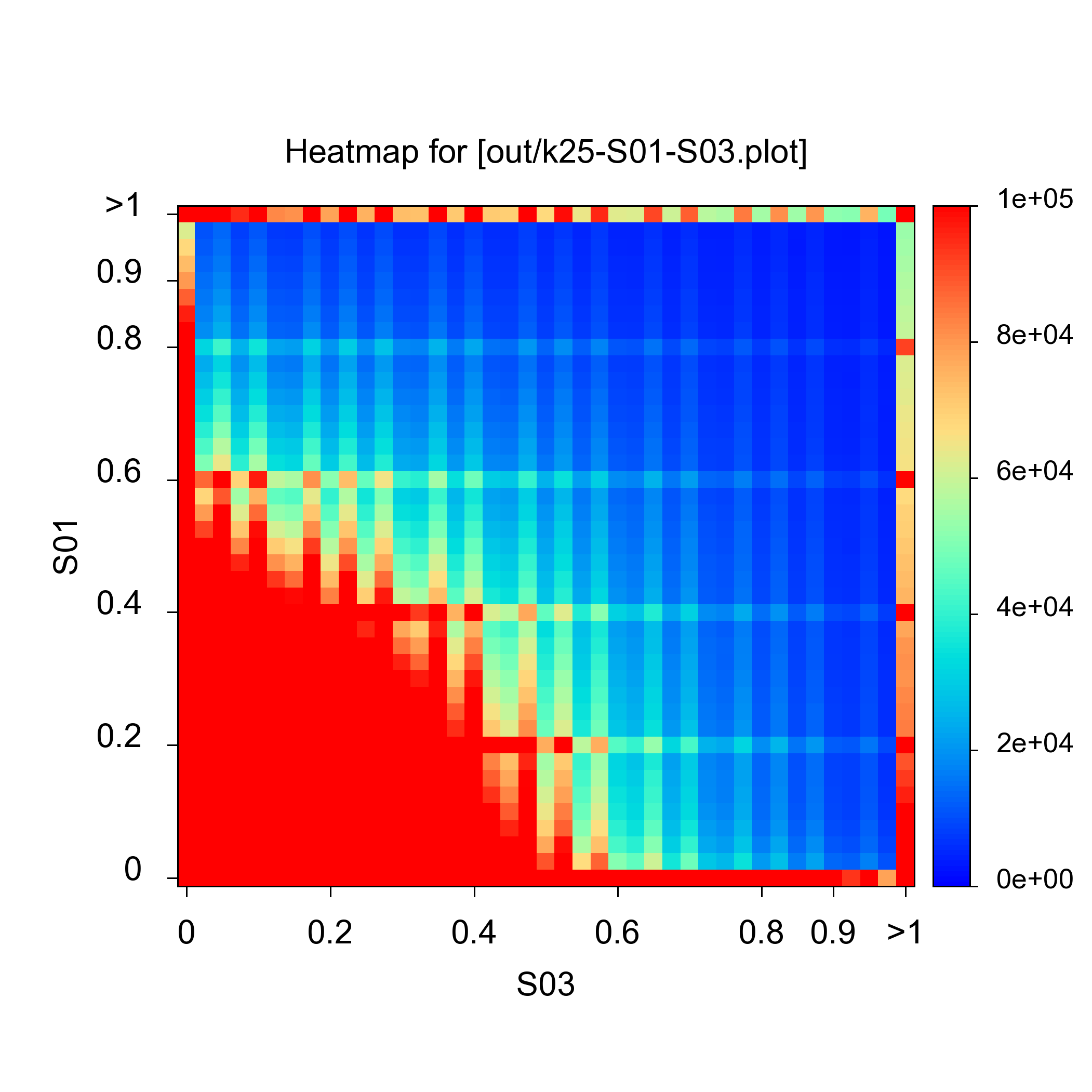

Supplement: S1 File — The joint distribution of K-mer frequency in the rest randomly paired samples. (ZIP) [file pone.0114520.s010.zip › Figure-S1/k25-S01-S03.plot.png]

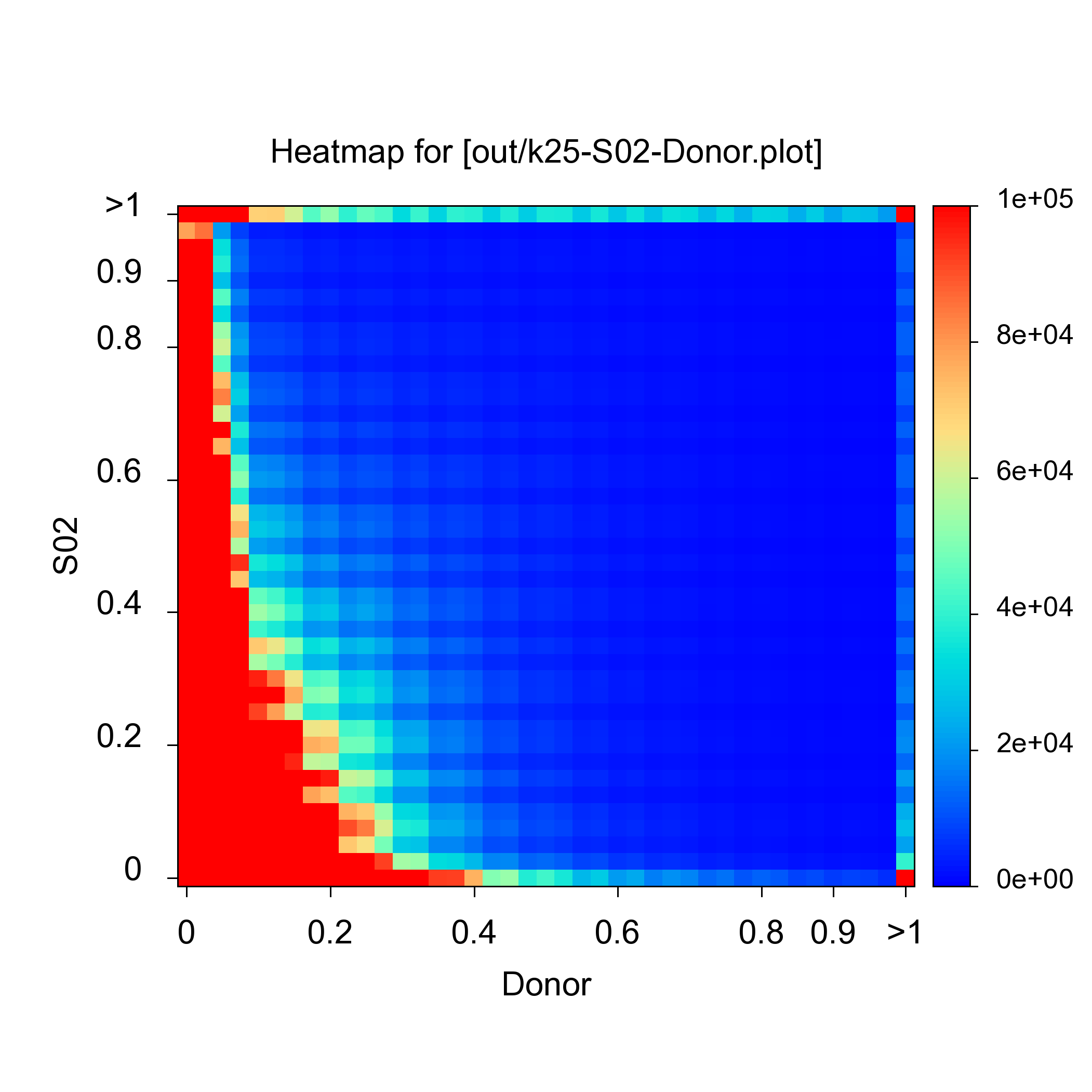

Supplement: S1 File — The joint distribution of K-mer frequency in the rest randomly paired samples. (ZIP) [file pone.0114520.s010.zip › Figure-S1/k25-S02-Donor.plot.png]

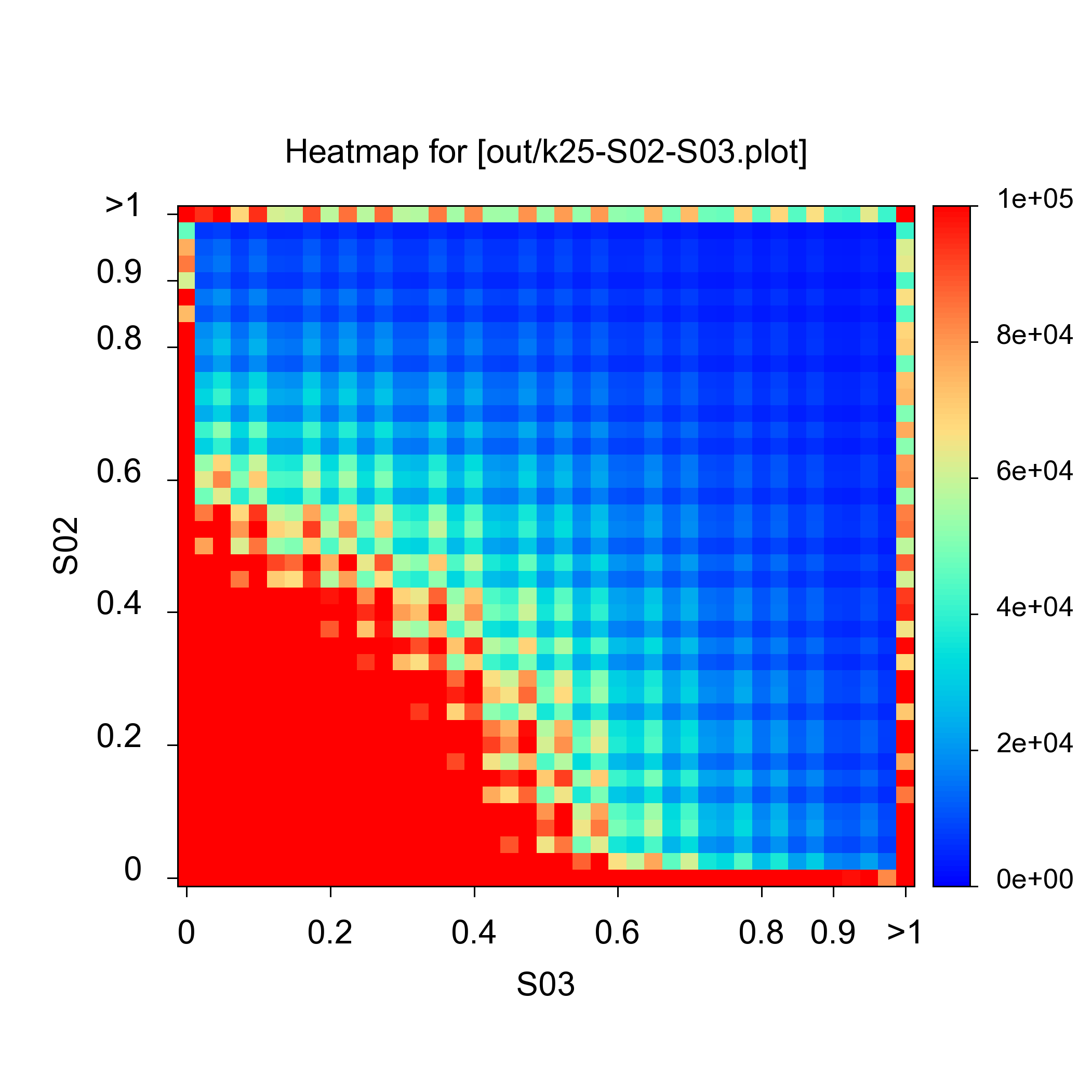

Supplement: S1 File — The joint distribution of K-mer frequency in the rest randomly paired samples. (ZIP) [file pone.0114520.s010.zip › Figure-S1/k25-S02-S03.plot.png]

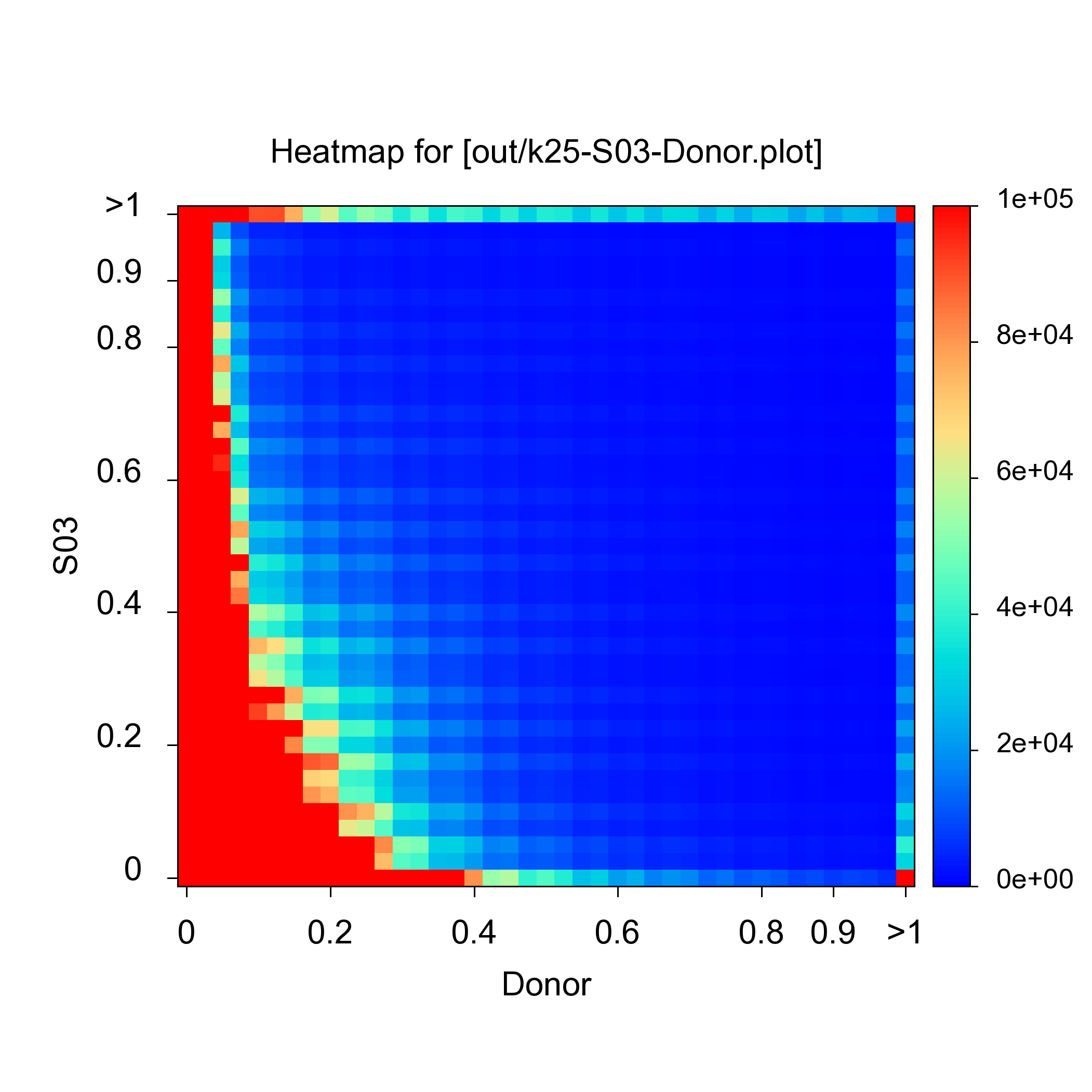

Supplement: S1 File — The joint distribution of K-mer frequency in the rest randomly paired samples. (ZIP) [file pone.0114520.s010.zip › Figure-S1/k25-S03-Donor.plot.png]

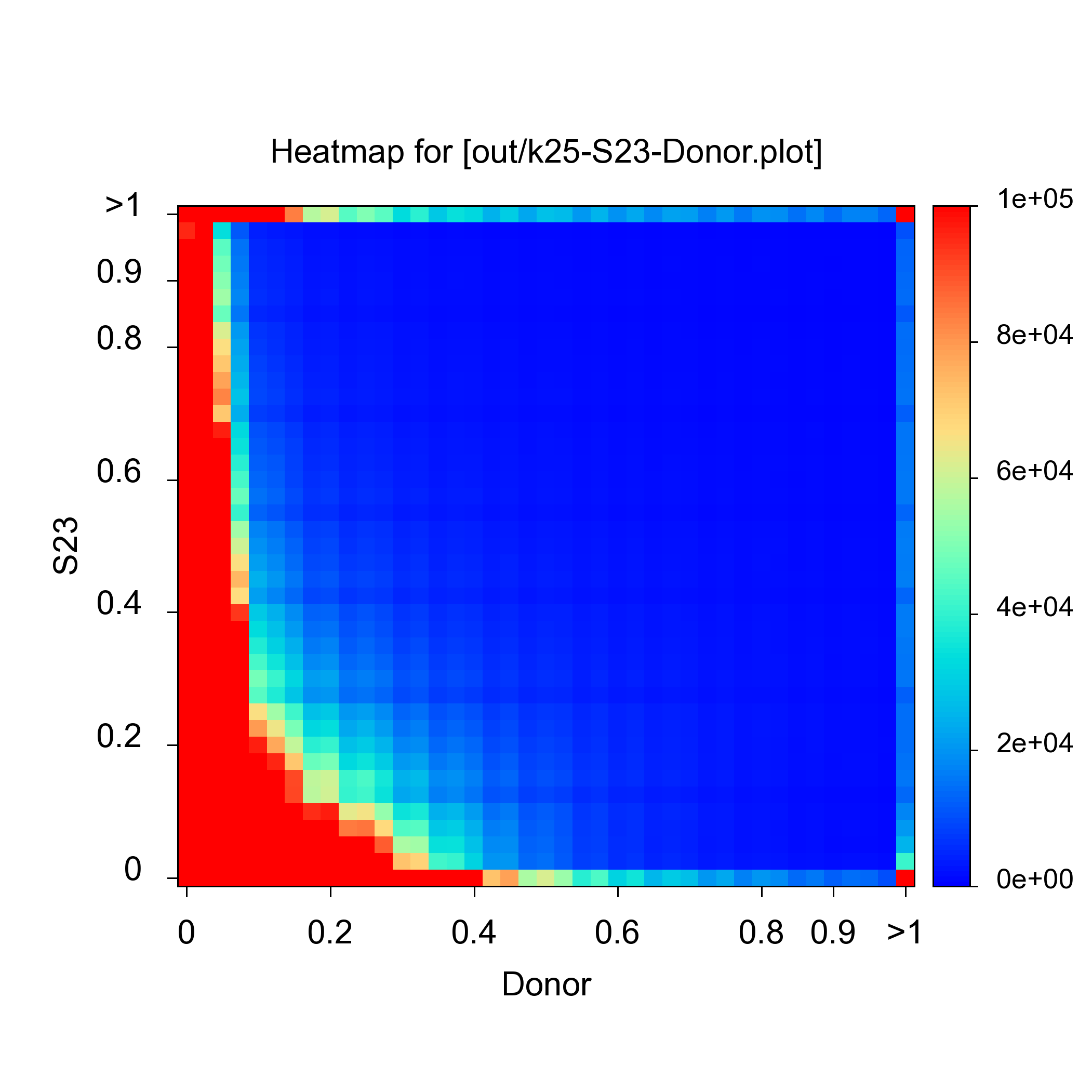

Supplement: S1 File — The joint distribution of K-mer frequency in the rest randomly paired samples. (ZIP) [file pone.0114520.s010.zip › Figure-S1/k25-S23-Donor.plot.png]

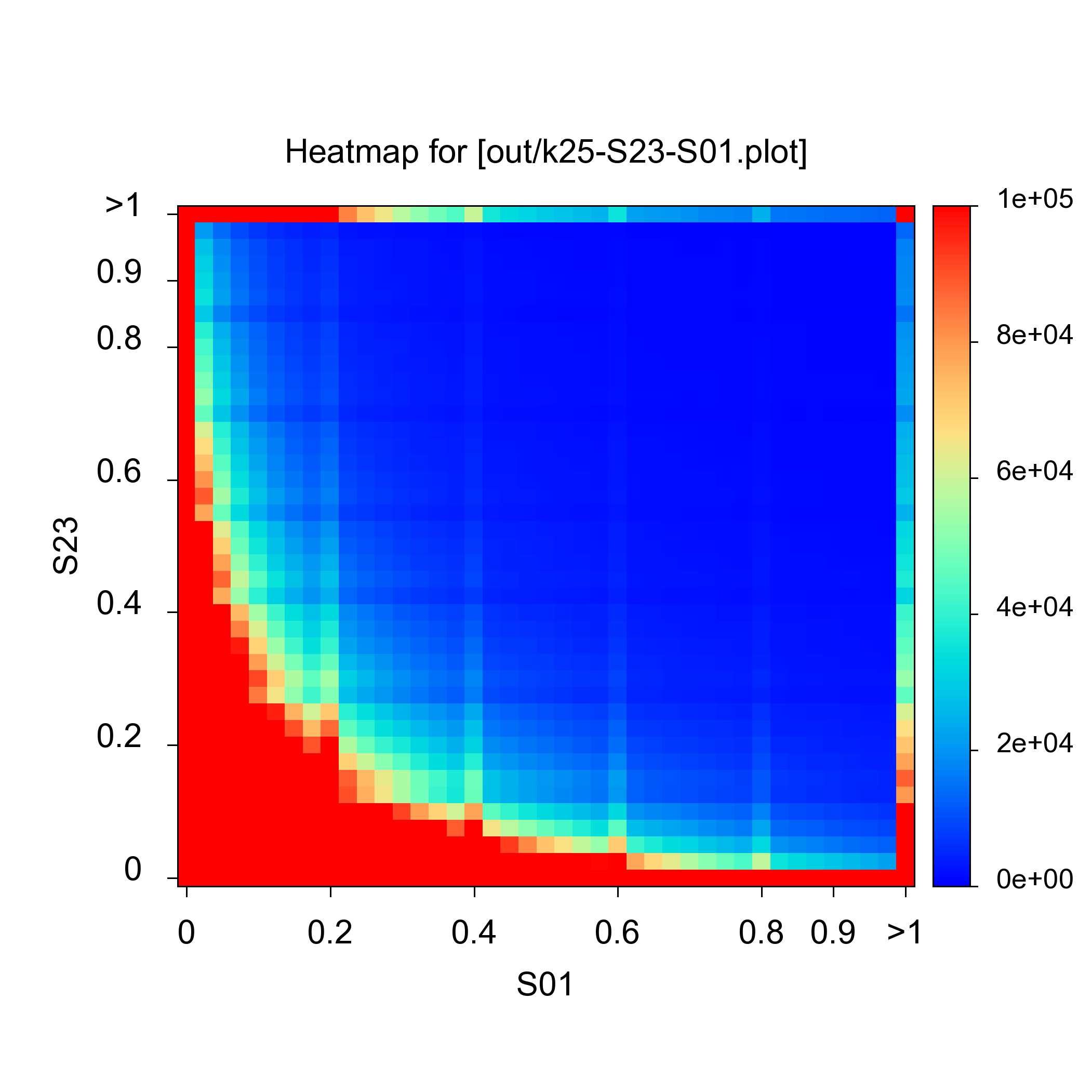

Supplement: S1 File — The joint distribution of K-mer frequency in the rest randomly paired samples. (ZIP) [file pone.0114520.s010.zip › Figure-S1/k25-S23-S01.plot.png]

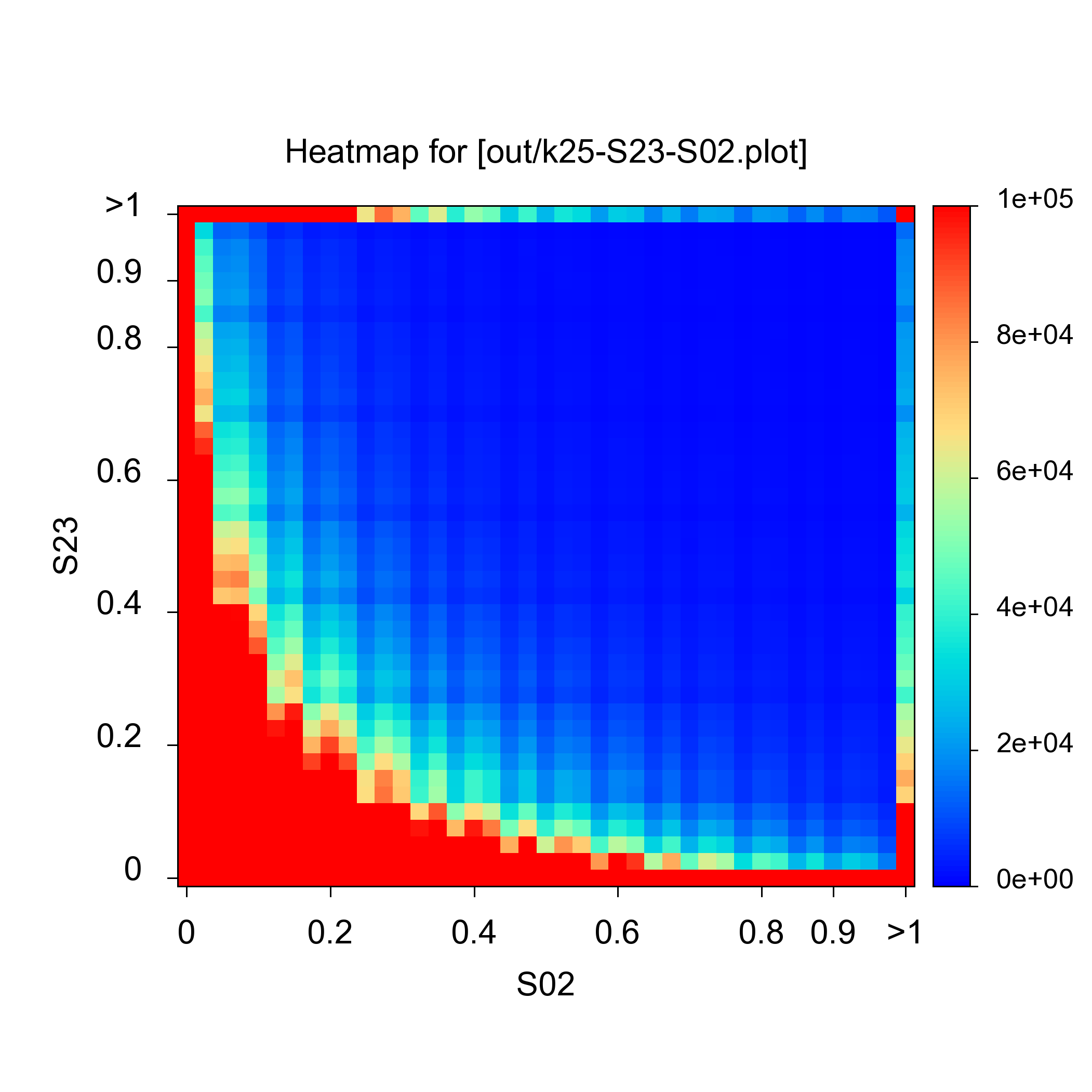

Supplement: S1 File — The joint distribution of K-mer frequency in the rest randomly paired samples. (ZIP) [file pone.0114520.s010.zip › Figure-S1/k25-S23-S02.plot.png]

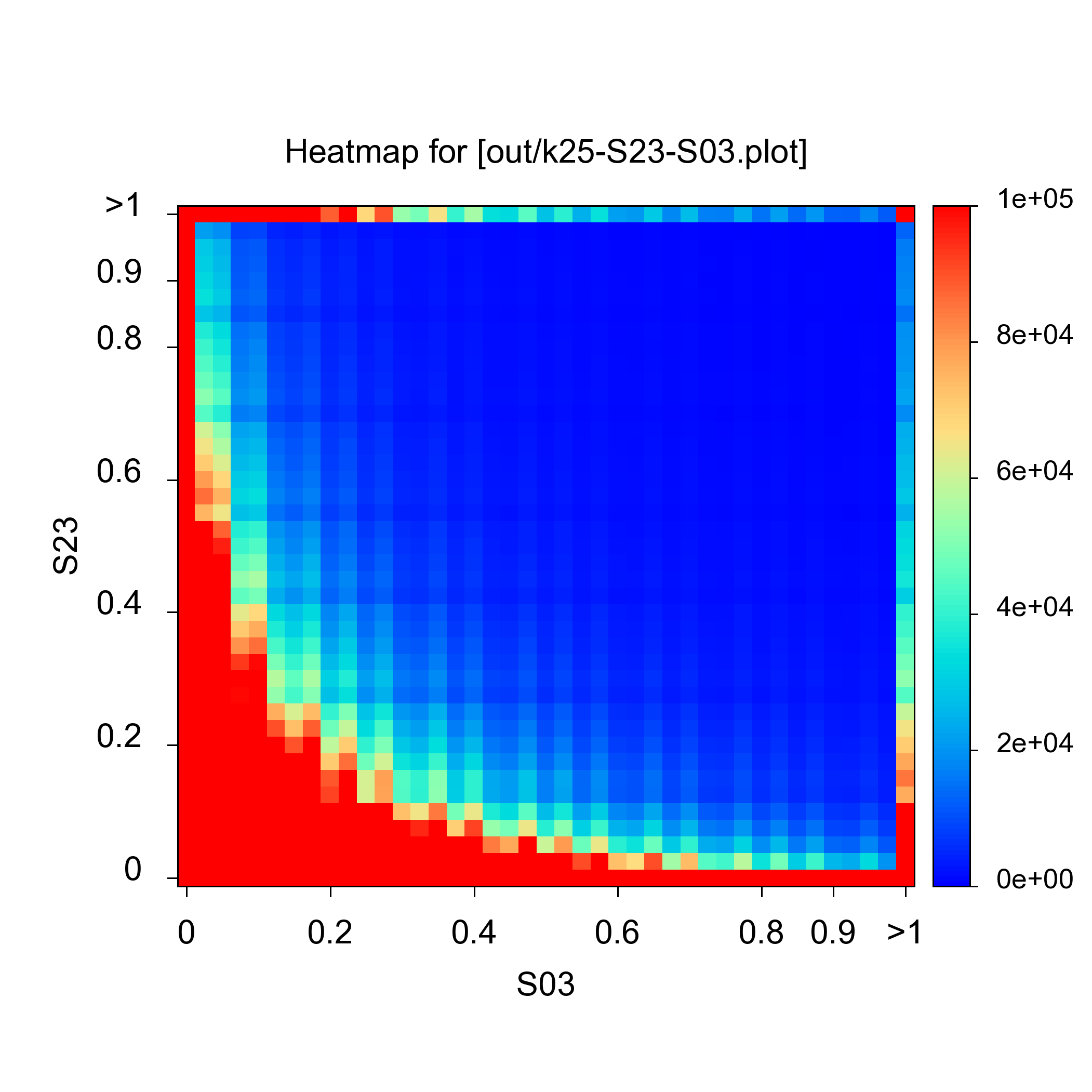

Supplement: S1 File — The joint distribution of K-mer frequency in the rest randomly paired samples. (ZIP) [file pone.0114520.s010.zip › Figure-S1/k25-S23-S03.plot.png]

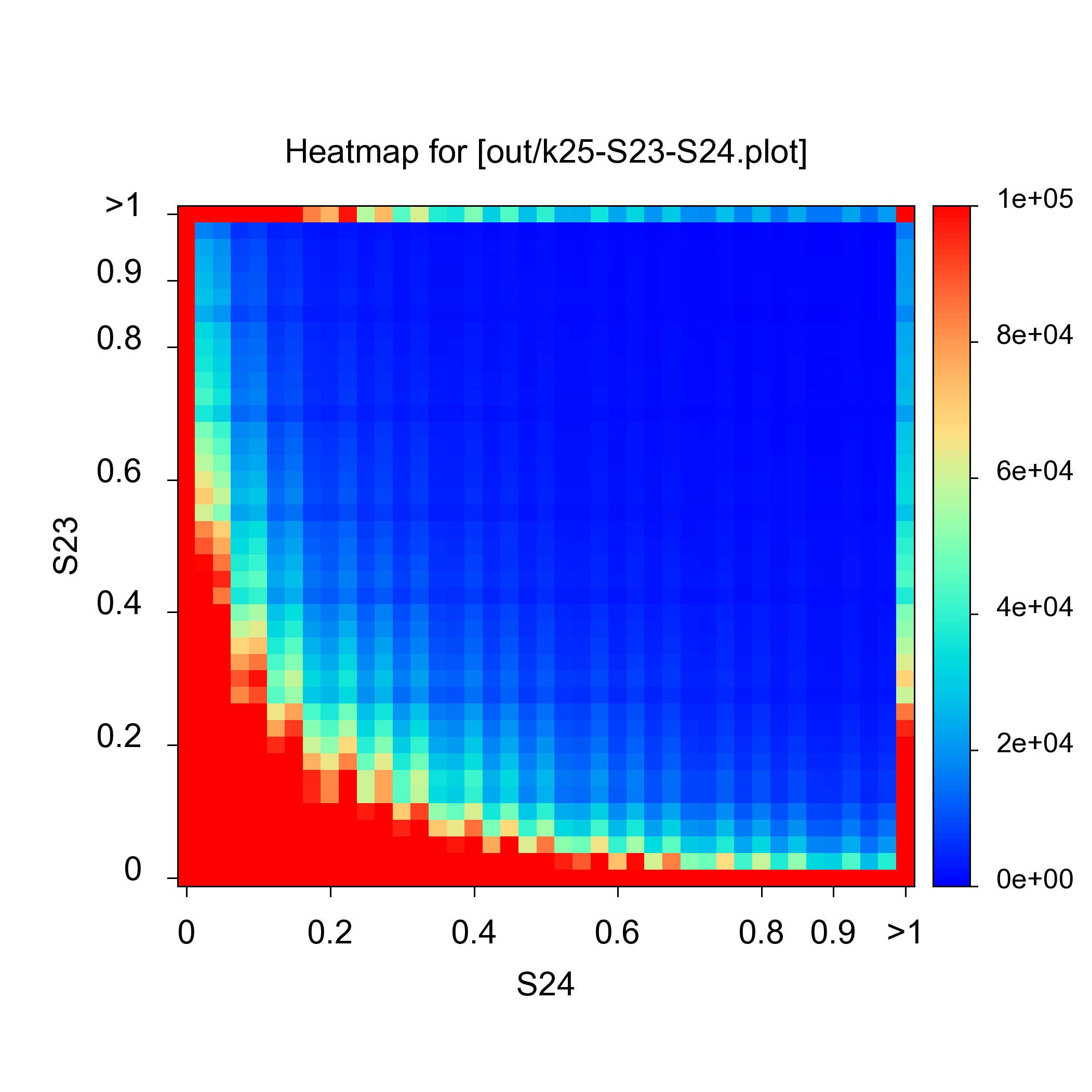

Supplement: S1 File — The joint distribution of K-mer frequency in the rest randomly paired samples. (ZIP) [file pone.0114520.s010.zip › Figure-S1/k25-S23-S24.plot.png]

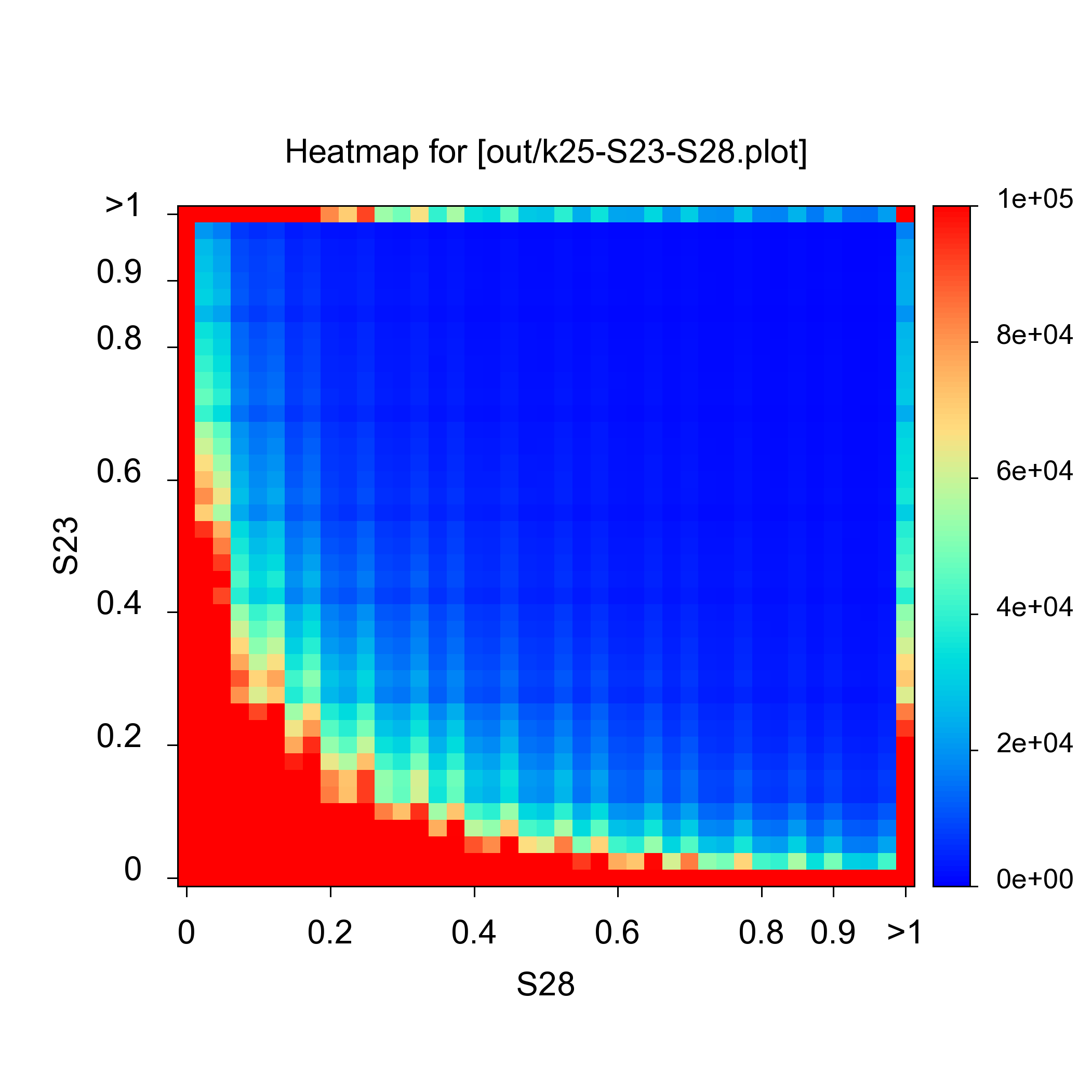

Supplement: S1 File — The joint distribution of K-mer frequency in the rest randomly paired samples. (ZIP) [file pone.0114520.s010.zip › Figure-S1/k25-S23-S28.plot.png]

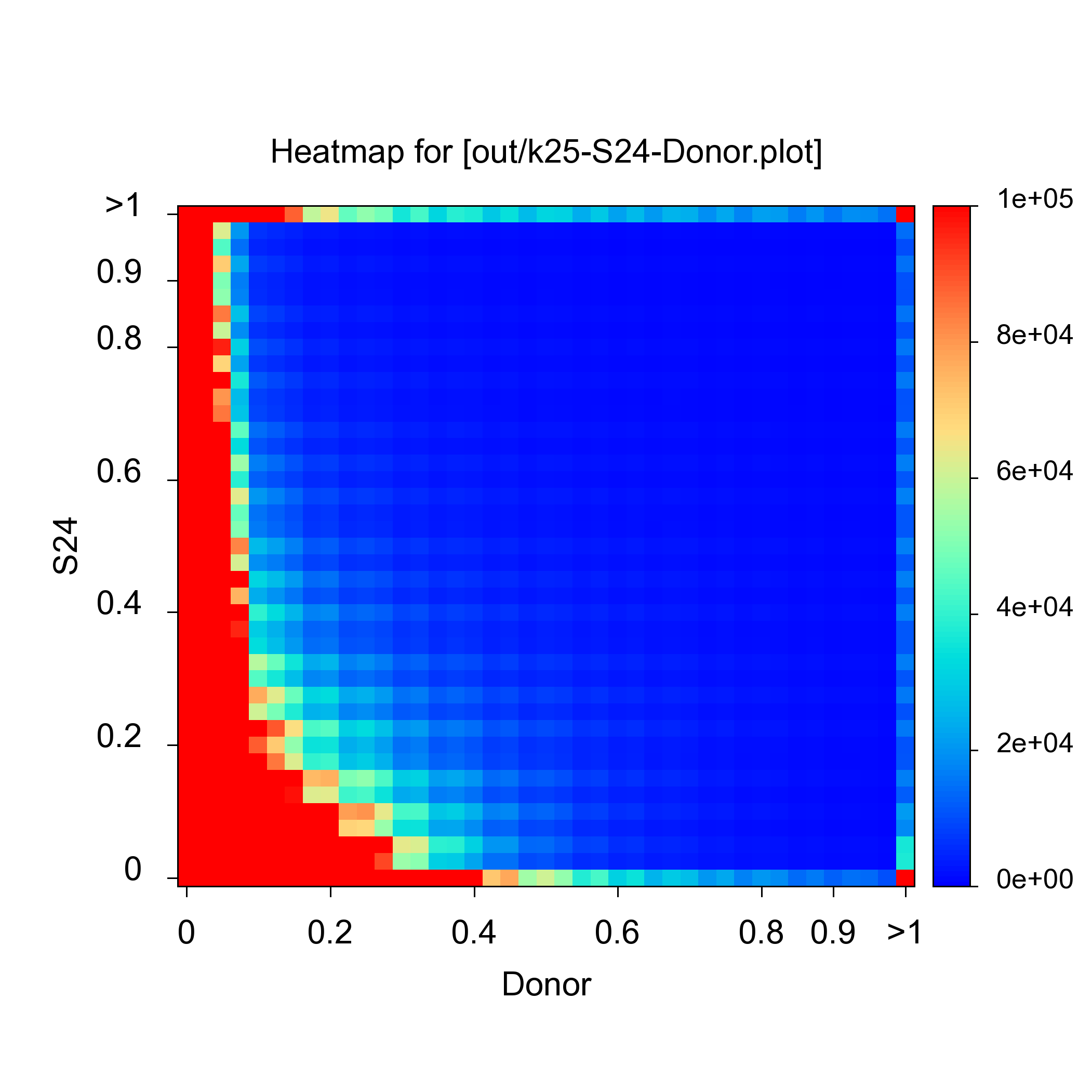

Supplement: S1 File — The joint distribution of K-mer frequency in the rest randomly paired samples. (ZIP) [file pone.0114520.s010.zip › Figure-S1/k25-S24-Donor.plot.png]

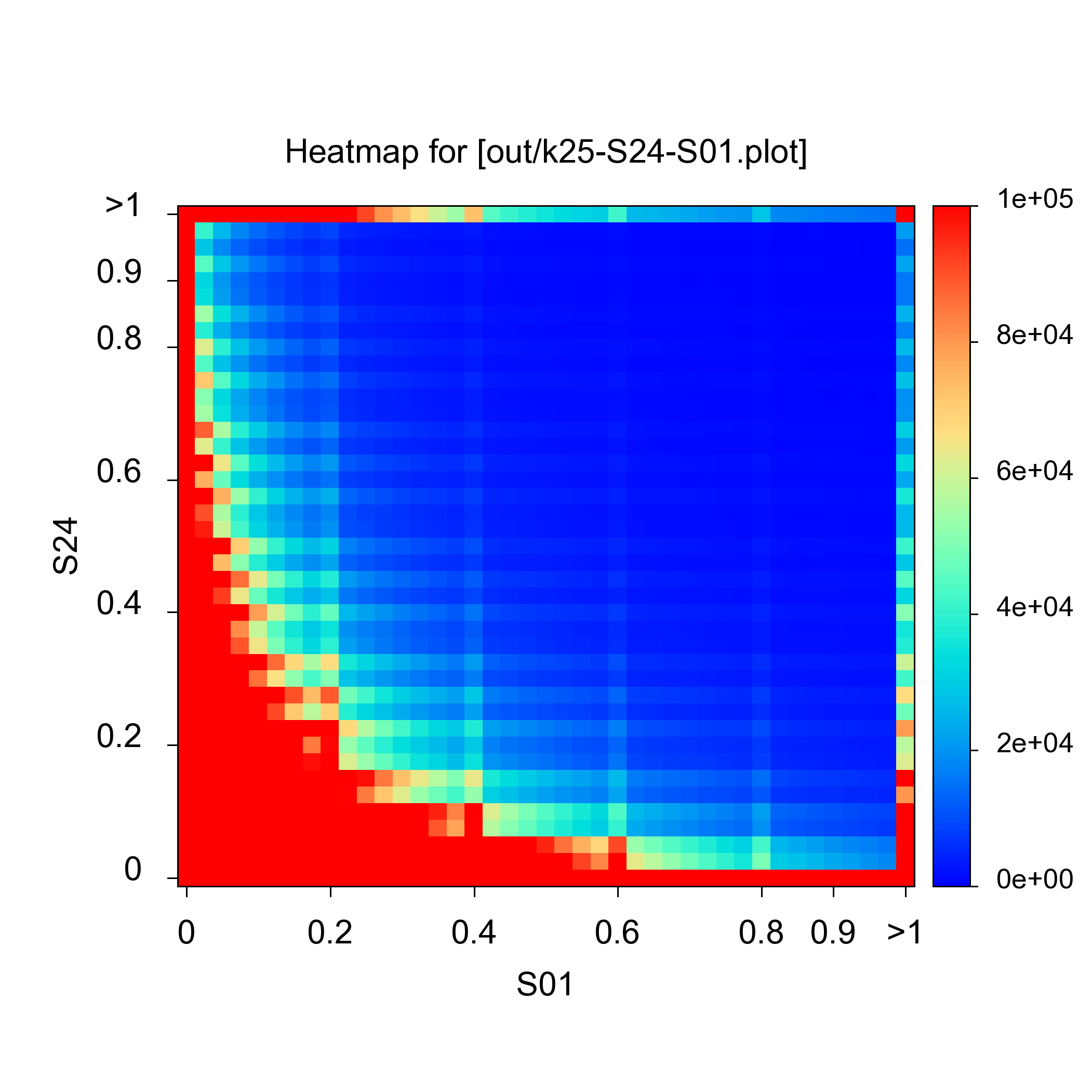

Supplement: S1 File — The joint distribution of K-mer frequency in the rest randomly paired samples. (ZIP) [file pone.0114520.s010.zip › Figure-S1/k25-S24-S01.plot.png]

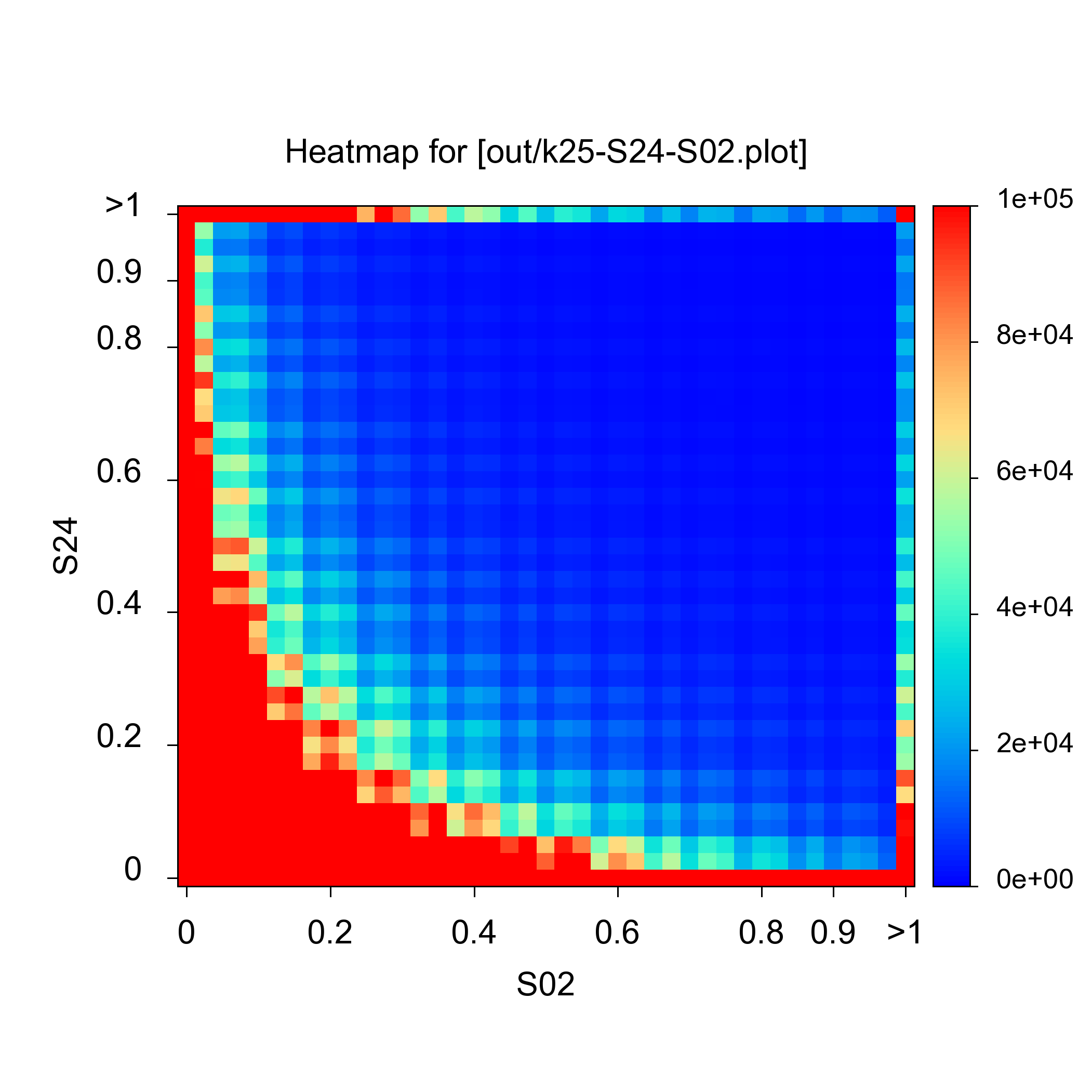

Supplement: S1 File — The joint distribution of K-mer frequency in the rest randomly paired samples. (ZIP) [file pone.0114520.s010.zip › Figure-S1/k25-S24-S02.plot.png]

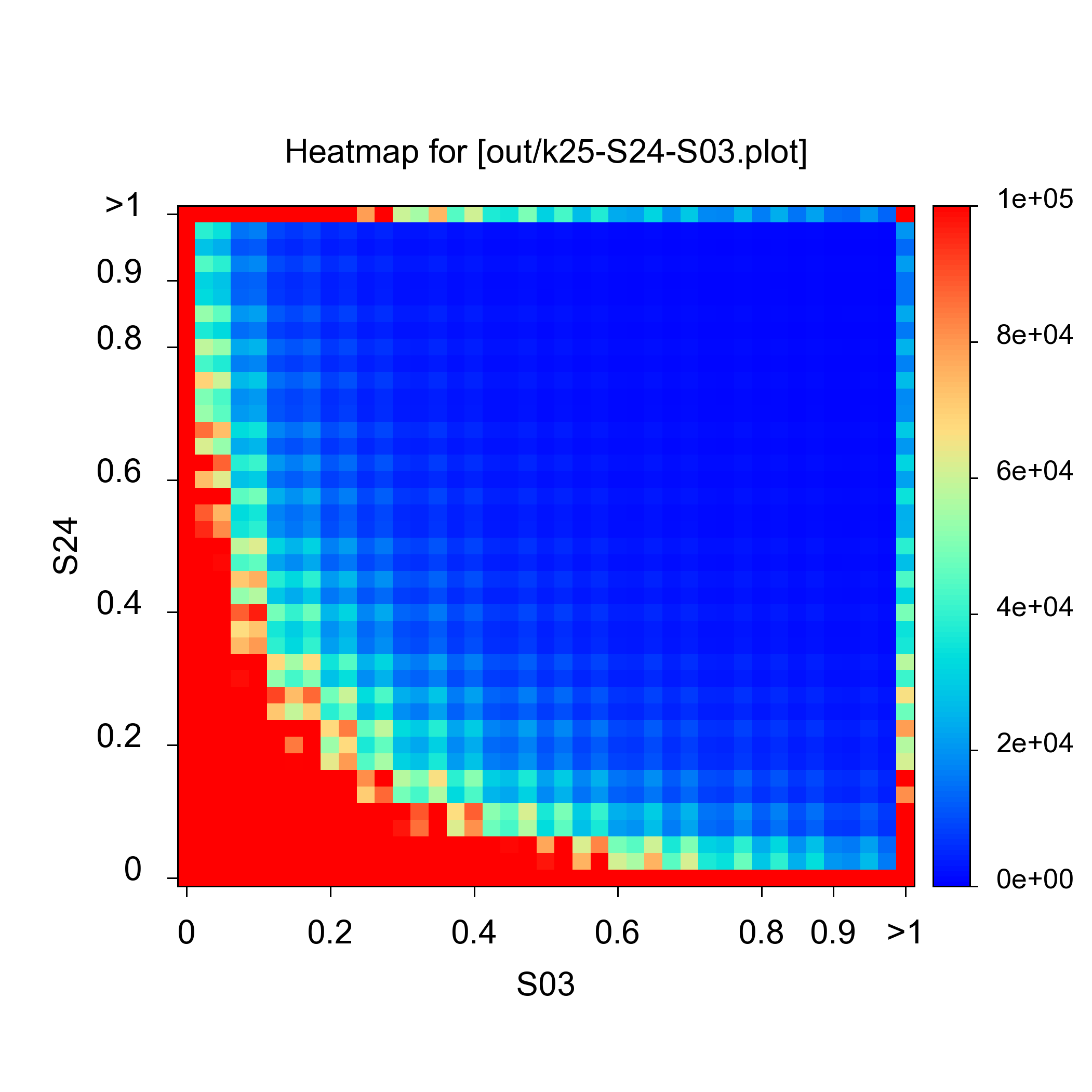

Supplement: S1 File — The joint distribution of K-mer frequency in the rest randomly paired samples. (ZIP) [file pone.0114520.s010.zip › Figure-S1/k25-S24-S03.plot.png]

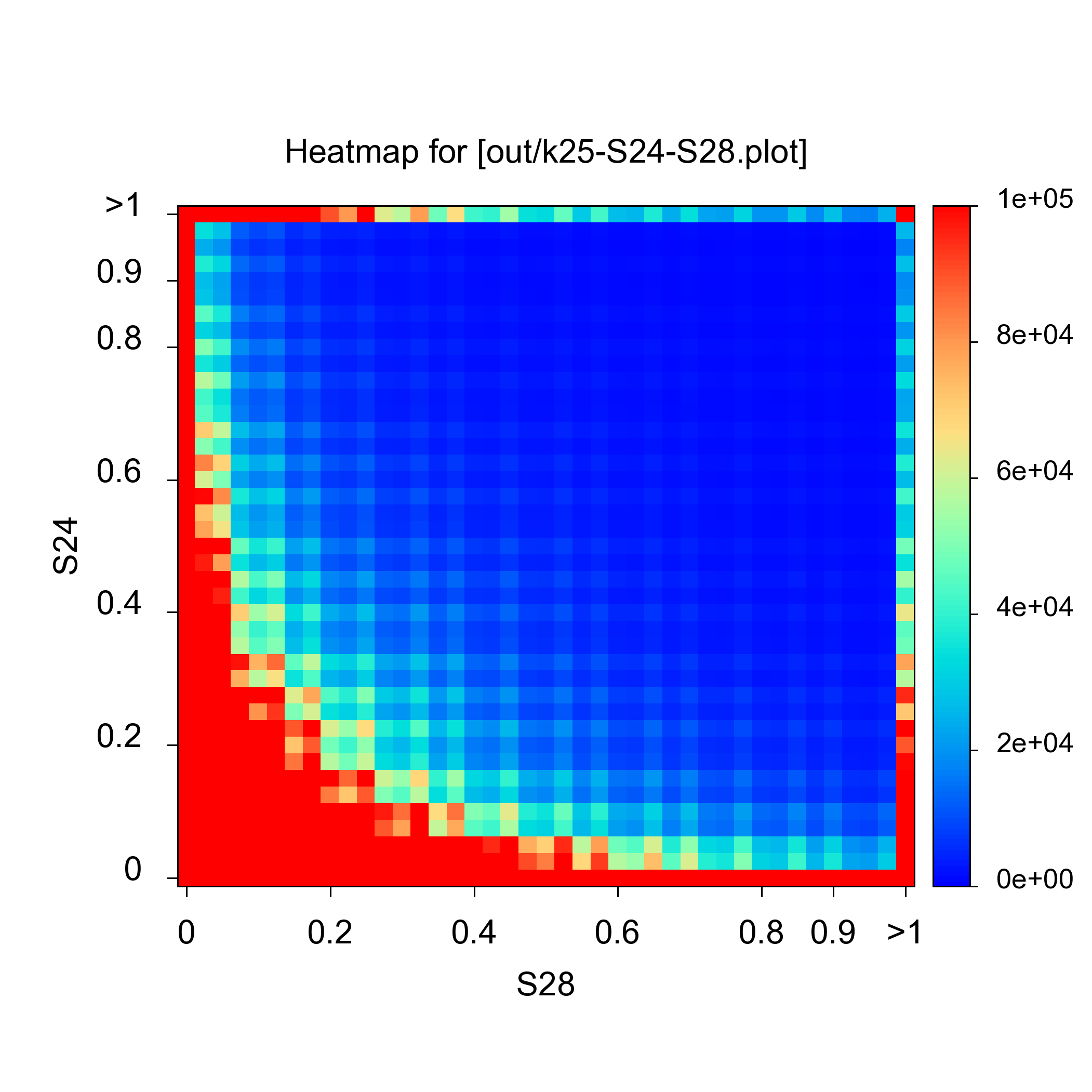

Supplement: S1 File — The joint distribution of K-mer frequency in the rest randomly paired samples. (ZIP) [file pone.0114520.s010.zip › Figure-S1/k25-S24-S28.plot.png]

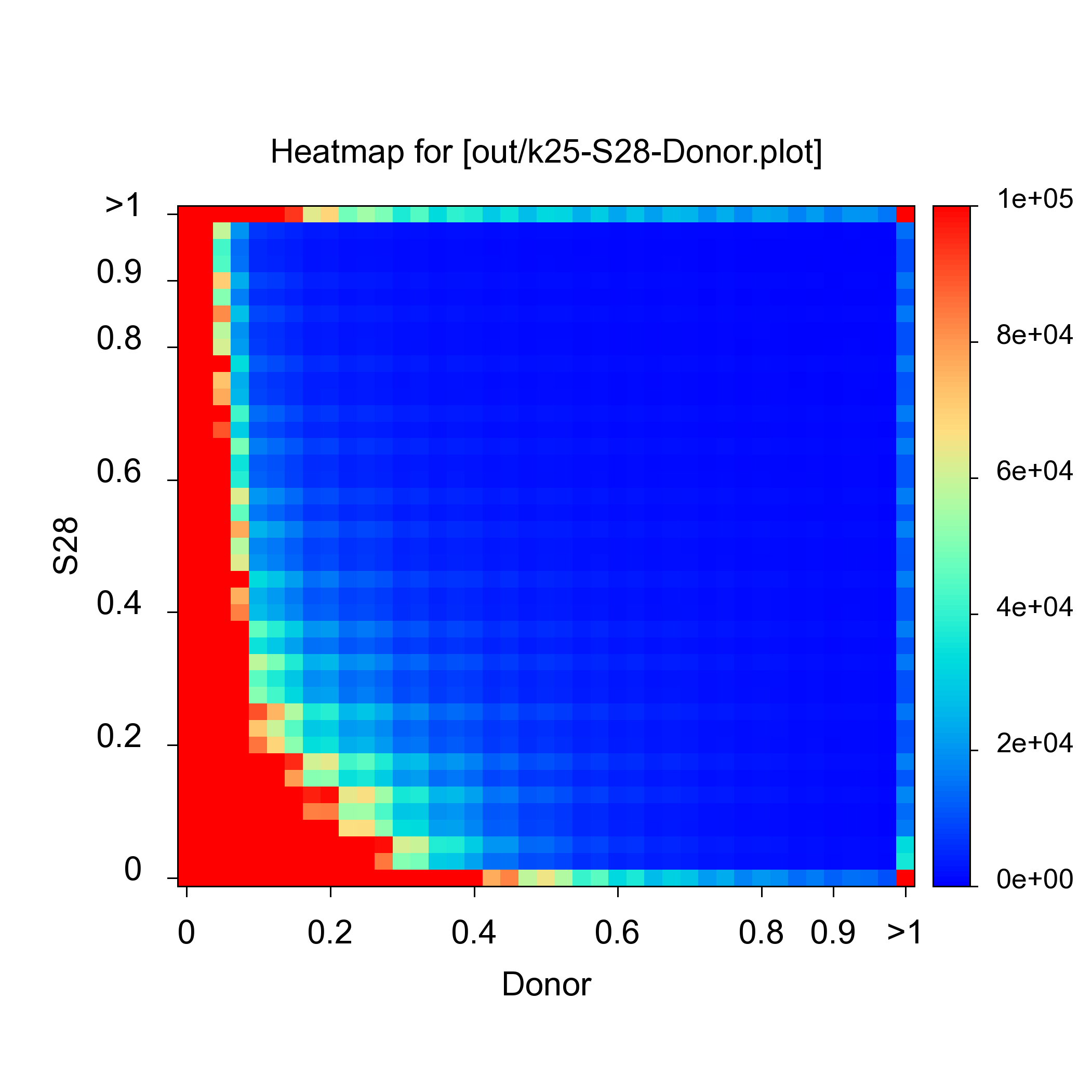

Supplement: S1 File — The joint distribution of K-mer frequency in the rest randomly paired samples. (ZIP) [file pone.0114520.s010.zip › Figure-S1/k25-S28-Donor.plot.png]

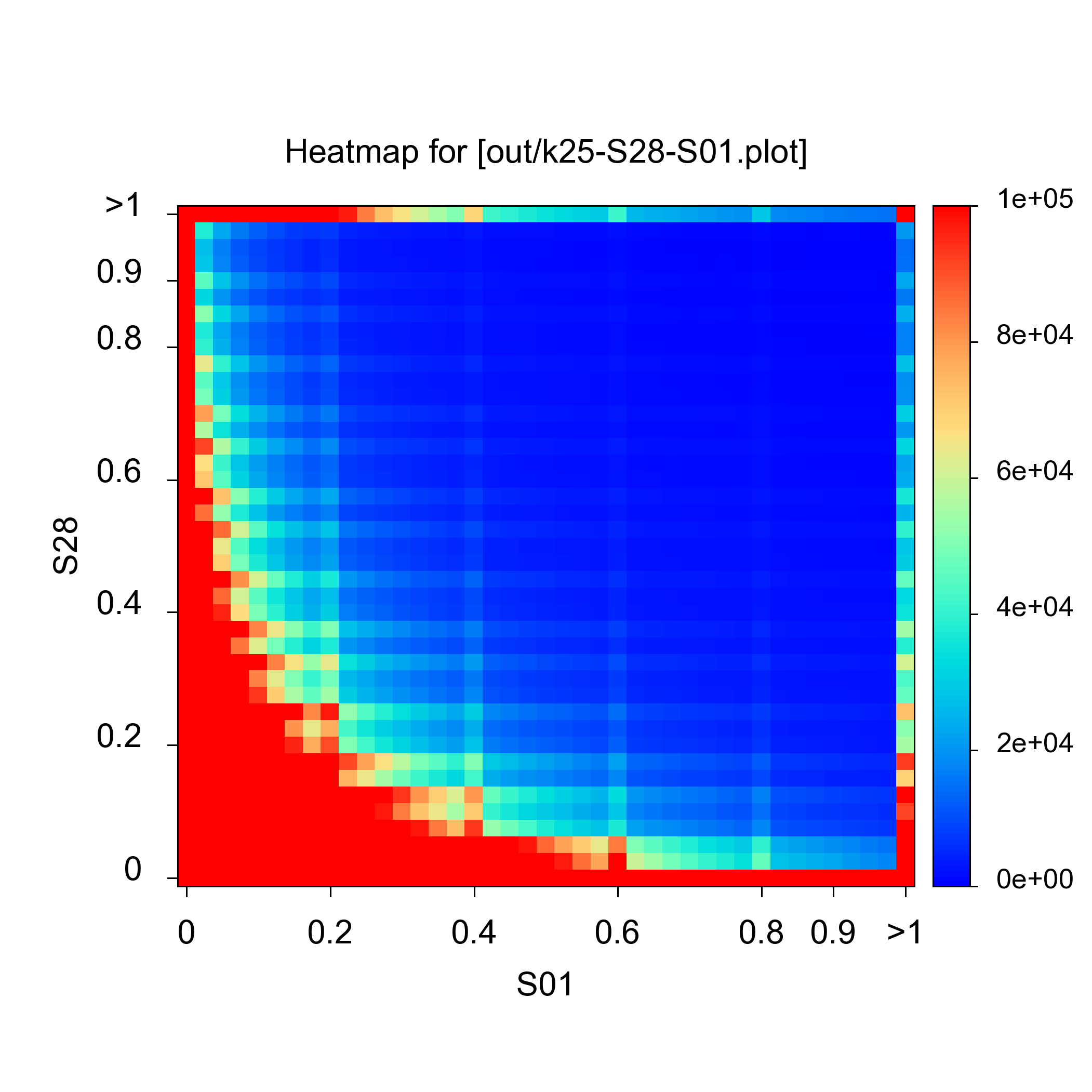

Supplement: S1 File — The joint distribution of K-mer frequency in the rest randomly paired samples. (ZIP) [file pone.0114520.s010.zip › Figure-S1/k25-S28-S01.plot.png]

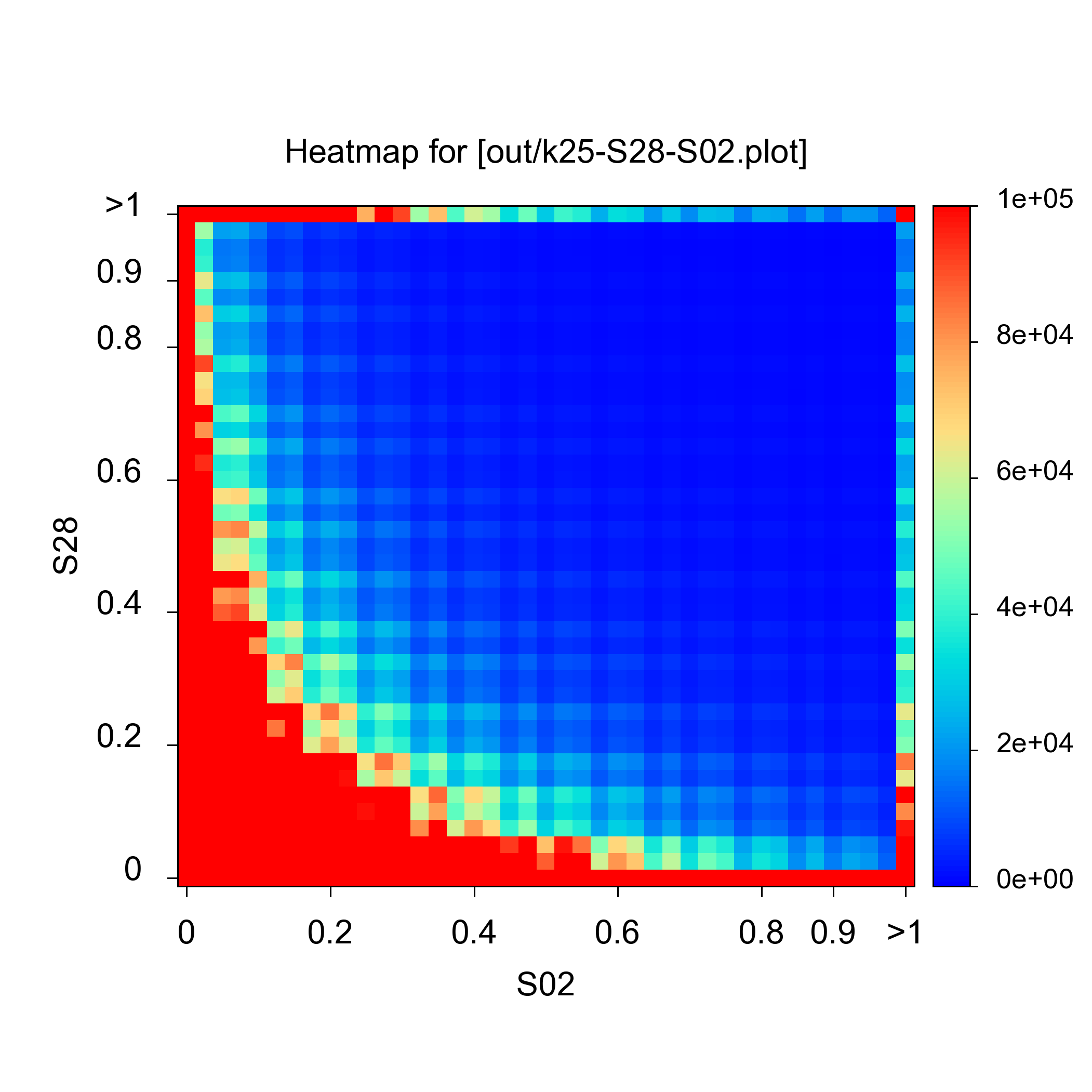

Supplement: S1 File — The joint distribution of K-mer frequency in the rest randomly paired samples. (ZIP) [file pone.0114520.s010.zip › Figure-S1/k25-S28-S02.plot.png]

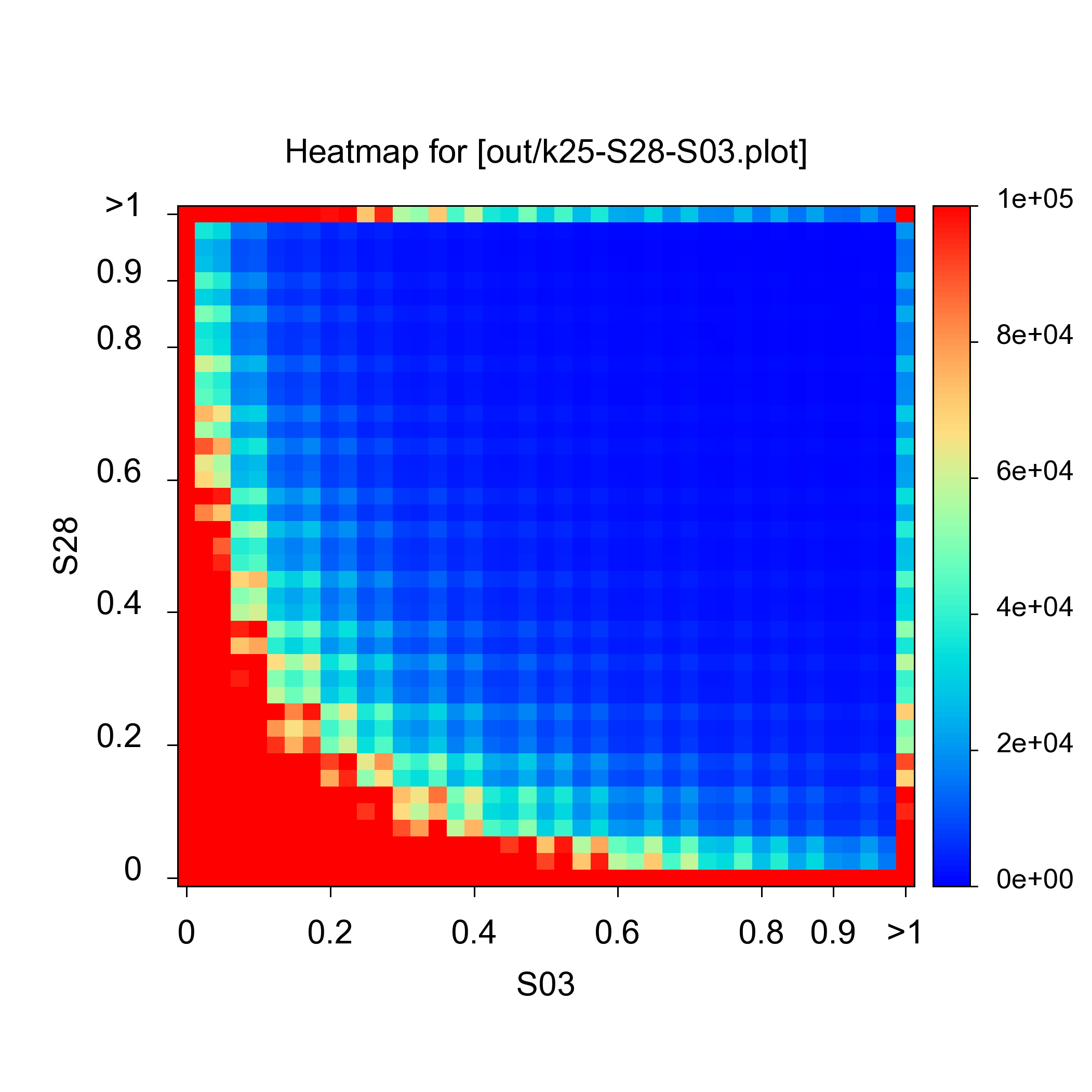

Supplement: S1 File — The joint distribution of K-mer frequency in the rest randomly paired samples. (ZIP) [file pone.0114520.s010.zip › Figure-S1/k25-S28-S03.plot.png]

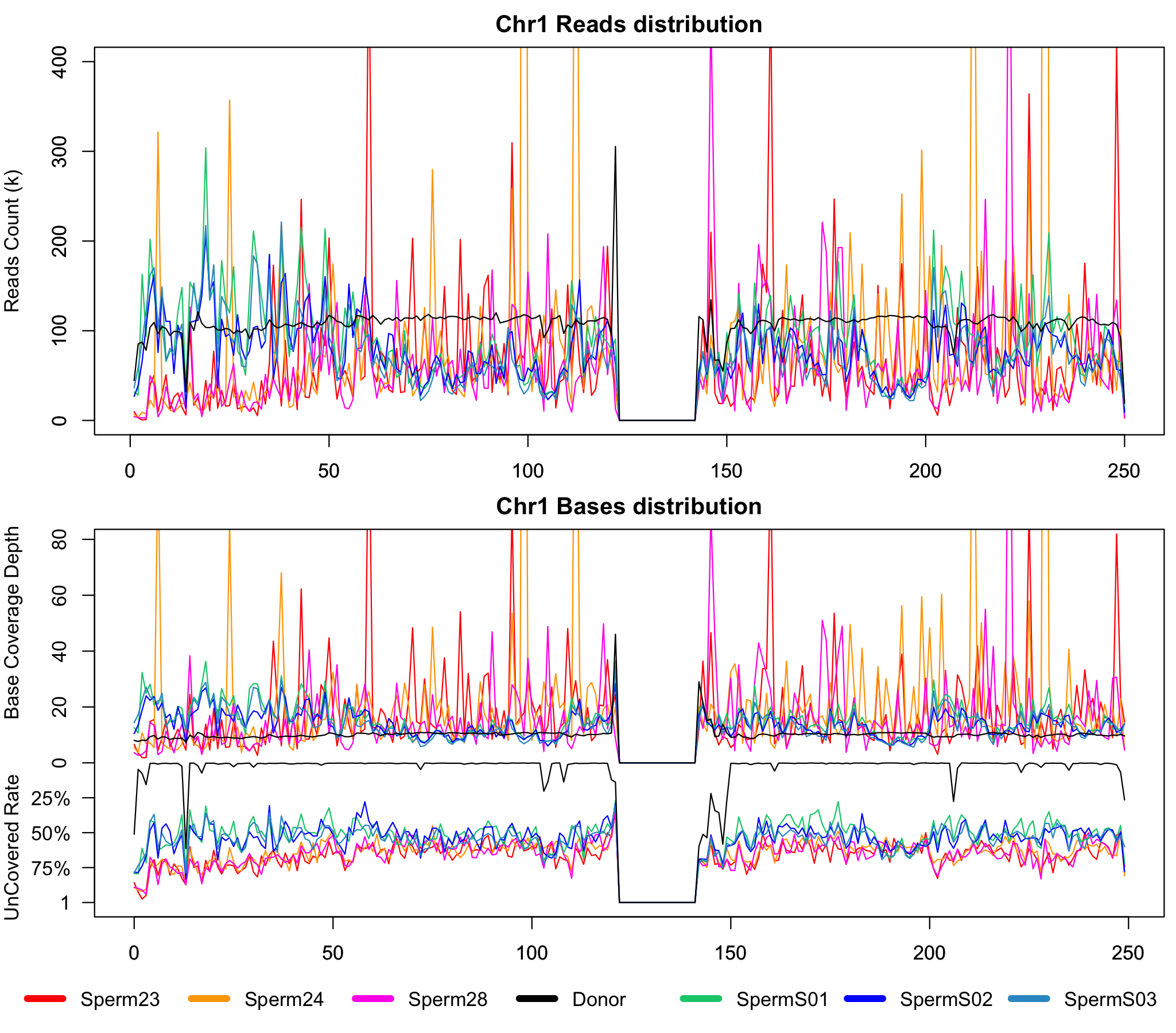

Supplement: S2 File — Genomic coverage on the rest chromosomes. Tilling window size is 1 M. (a) Reads counts in each window. (b) Base coverage depth (upper) and uncovered base rate (lower) in each window. Sperm 23∼28 are MDA samples and Sperm S01∼S03 are MALBAC samples. (ZIP) [file pone.0114520.s011.zip › Figure-S3/CoveragePlot.chr01.png]

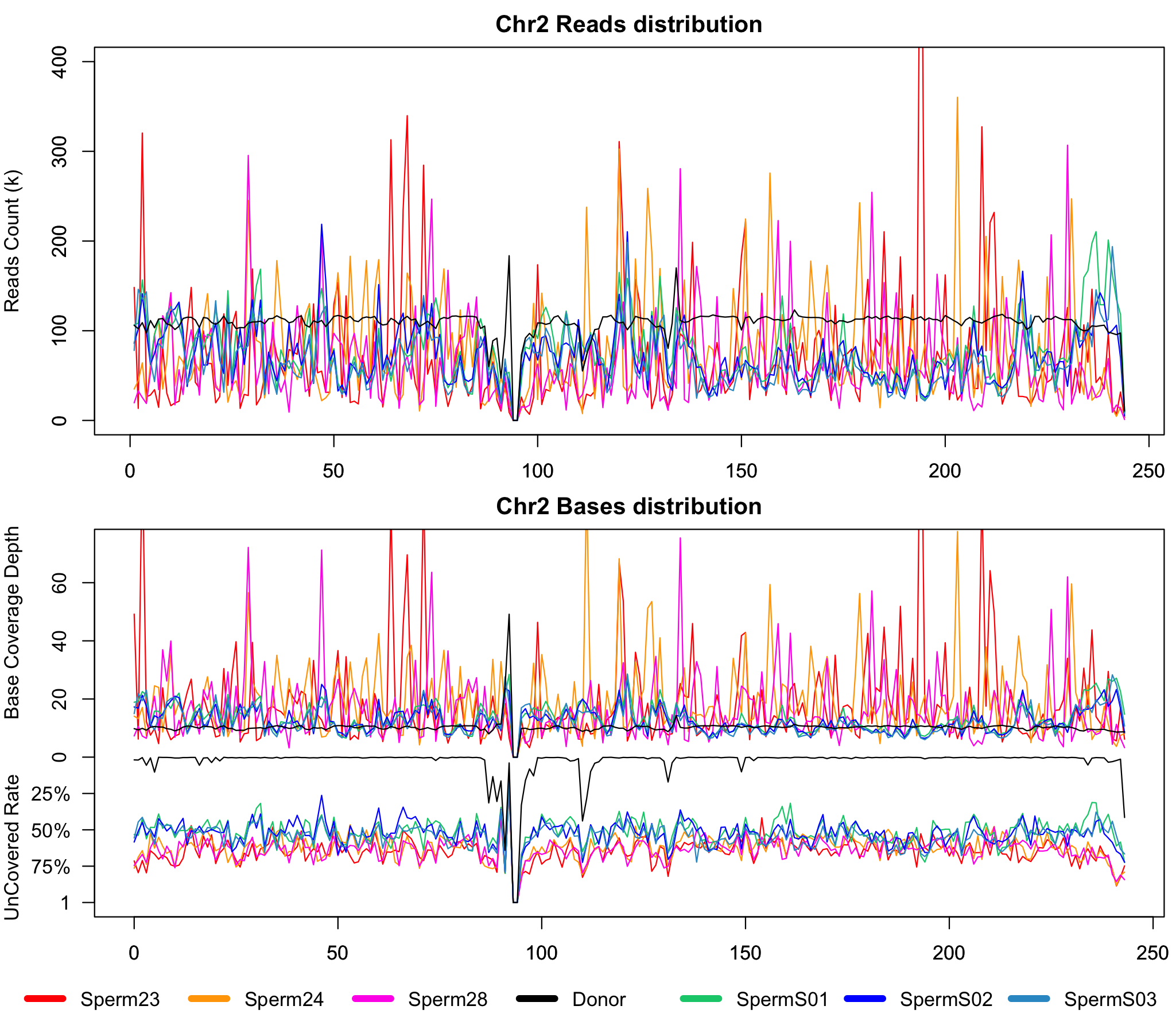

Supplement: S2 File — Genomic coverage on the rest chromosomes. Tilling window size is 1 M. (a) Reads counts in each window. (b) Base coverage depth (upper) and uncovered base rate (lower) in each window. Sperm 23∼28 are MDA samples and Sperm S01∼S03 are MALBAC samples. (ZIP) [file pone.0114520.s011.zip › Figure-S3/CoveragePlot.chr02.png]

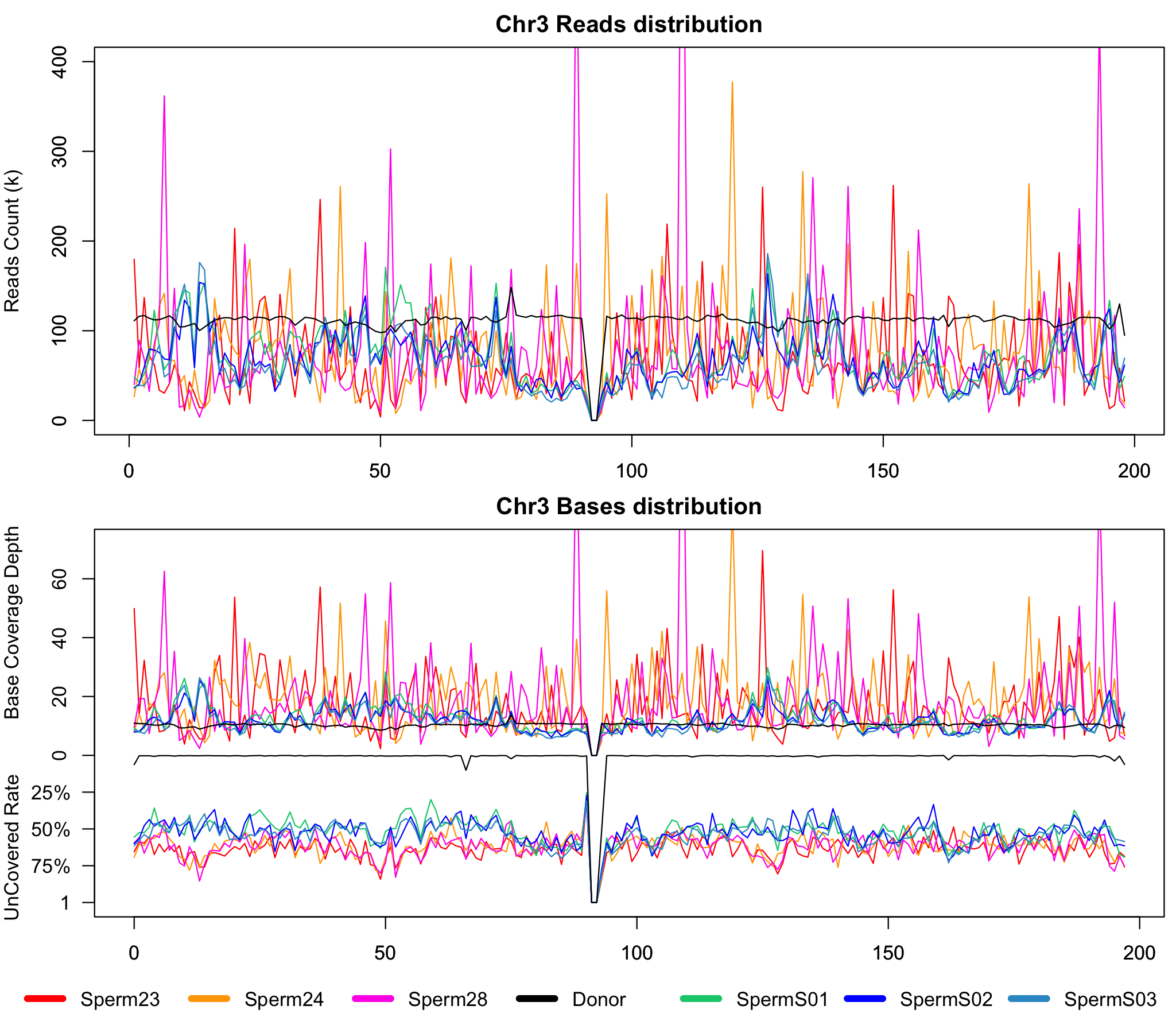

Supplement: S2 File — Genomic coverage on the rest chromosomes. Tilling window size is 1 M. (a) Reads counts in each window. (b) Base coverage depth (upper) and uncovered base rate (lower) in each window. Sperm 23∼28 are MDA samples and Sperm S01∼S03 are MALBAC samples. (ZIP) [file pone.0114520.s011.zip › Figure-S3/CoveragePlot.chr03.png]

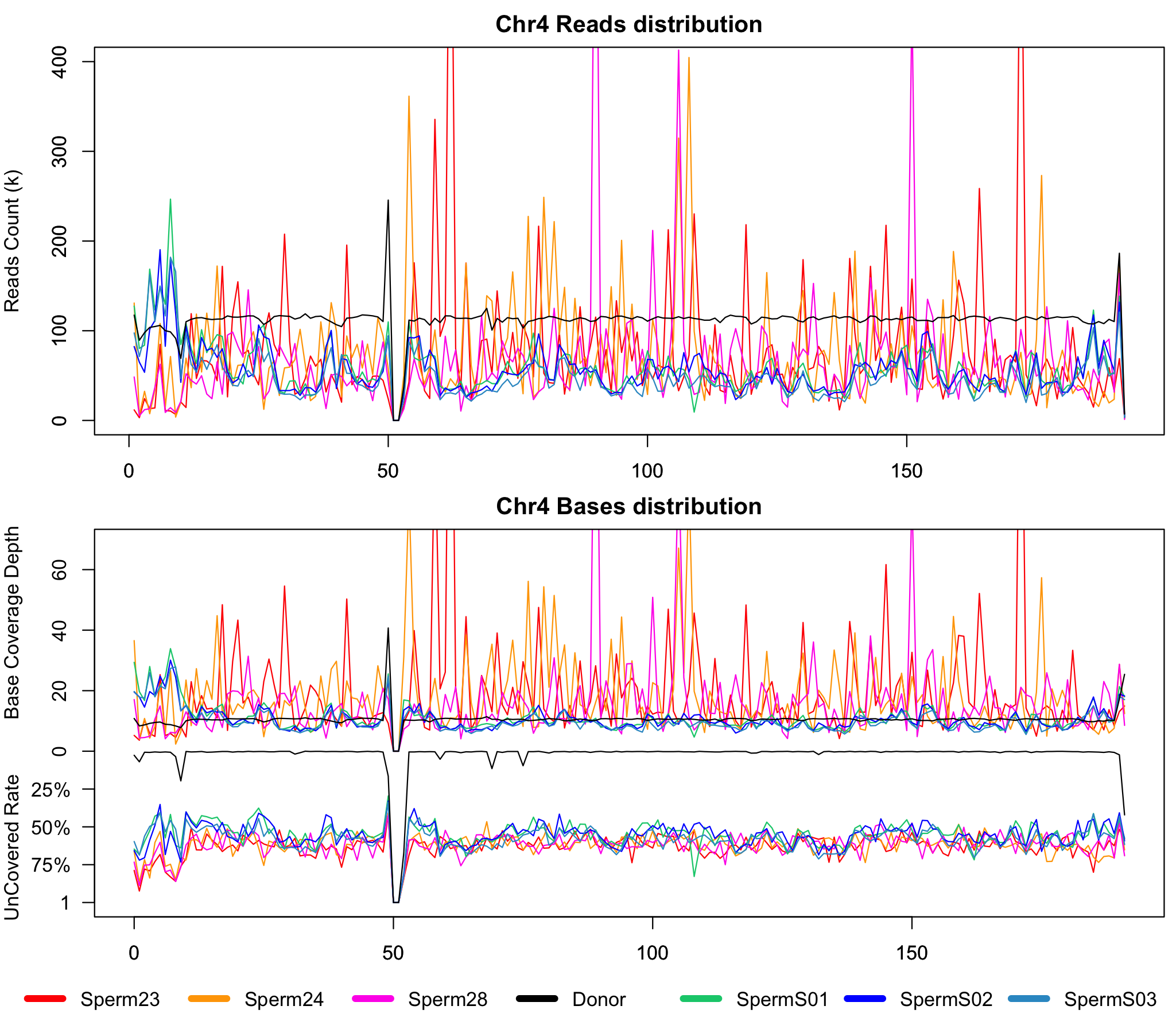

Supplement: S2 File — Genomic coverage on the rest chromosomes. Tilling window size is 1 M. (a) Reads counts in each window. (b) Base coverage depth (upper) and uncovered base rate (lower) in each window. Sperm 23∼28 are MDA samples and Sperm S01∼S03 are MALBAC samples. (ZIP) [file pone.0114520.s011.zip › Figure-S3/CoveragePlot.chr04.png]

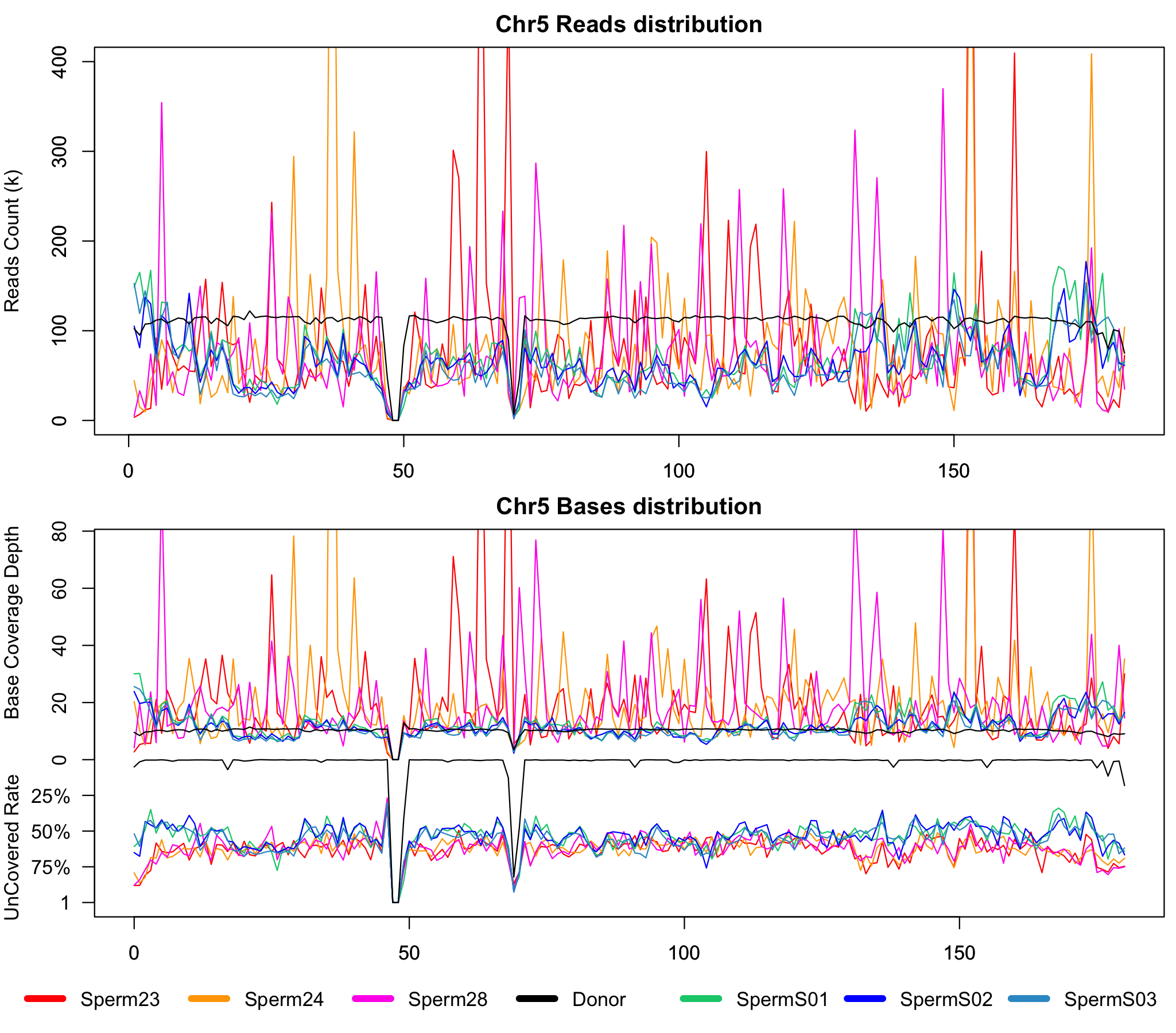

Supplement: S2 File — Genomic coverage on the rest chromosomes. Tilling window size is 1 M. (a) Reads counts in each window. (b) Base coverage depth (upper) and uncovered base rate (lower) in each window. Sperm 23∼28 are MDA samples and Sperm S01∼S03 are MALBAC samples. (ZIP) [file pone.0114520.s011.zip › Figure-S3/CoveragePlot.chr05.png]

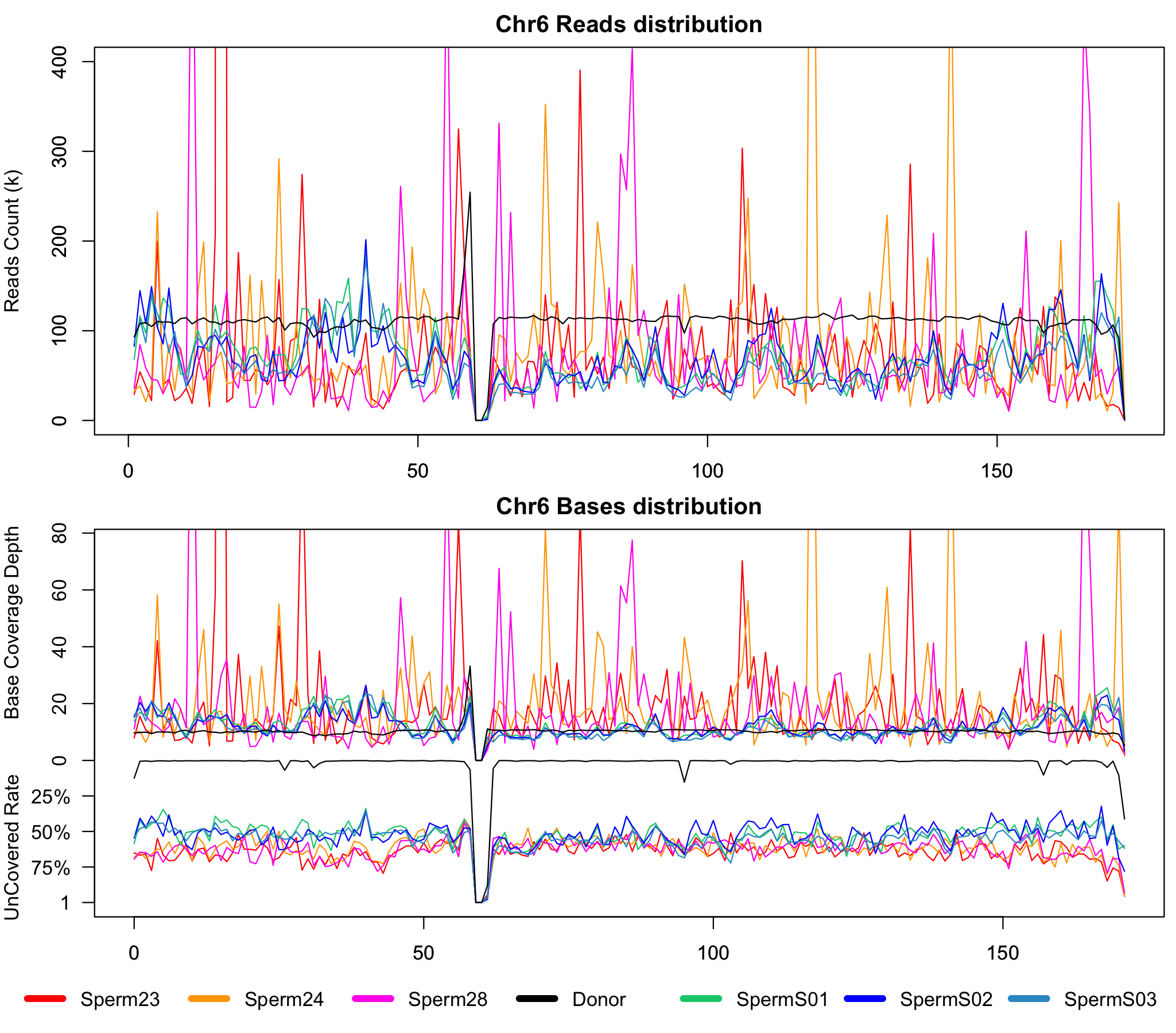

Supplement: S2 File — Genomic coverage on the rest chromosomes. Tilling window size is 1 M. (a) Reads counts in each window. (b) Base coverage depth (upper) and uncovered base rate (lower) in each window. Sperm 23∼28 are MDA samples and Sperm S01∼S03 are MALBAC samples. (ZIP) [file pone.0114520.s011.zip › Figure-S3/CoveragePlot.chr06.png]

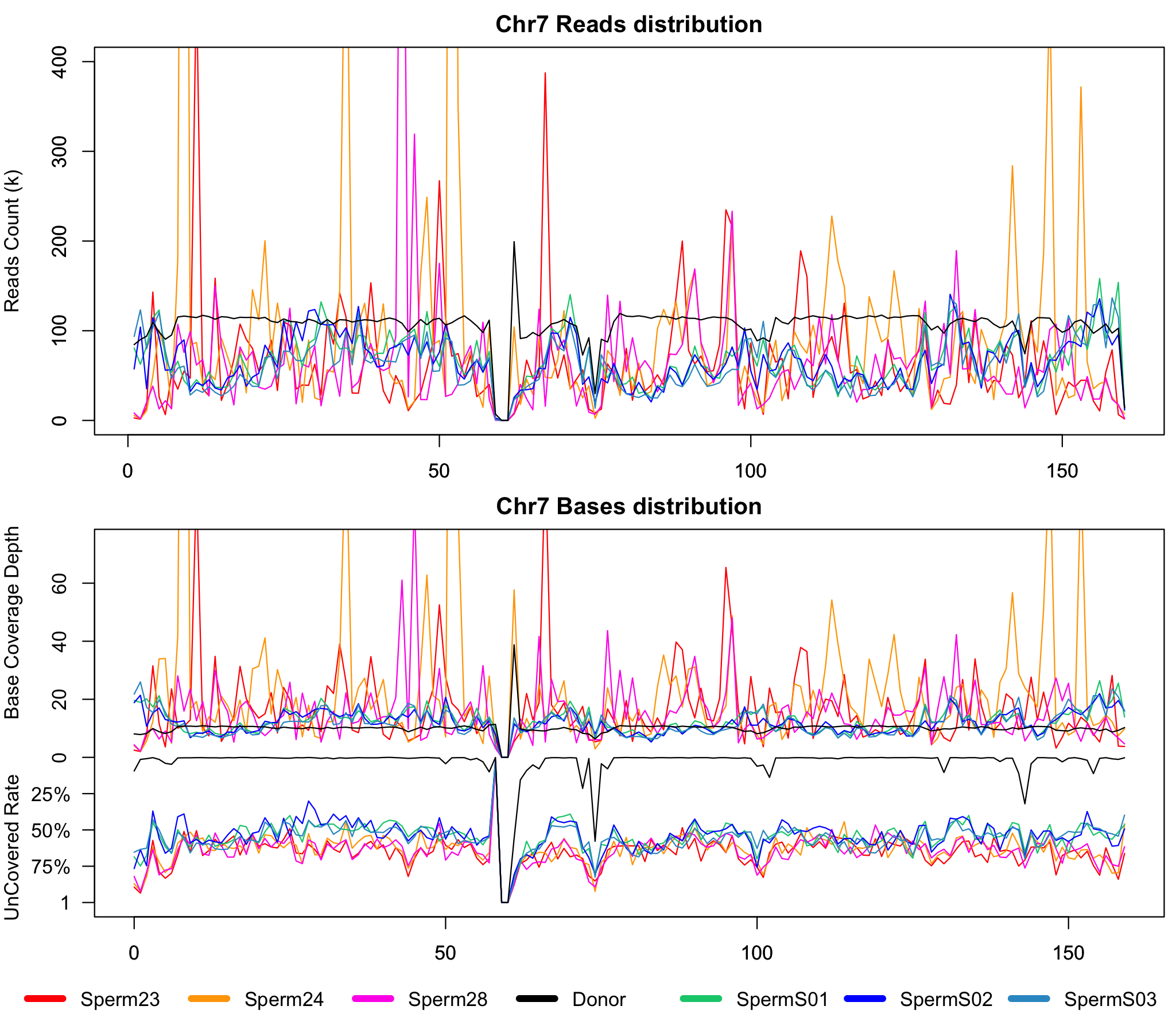

Supplement: S2 File — Genomic coverage on the rest chromosomes. Tilling window size is 1 M. (a) Reads counts in each window. (b) Base coverage depth (upper) and uncovered base rate (lower) in each window. Sperm 23∼28 are MDA samples and Sperm S01∼S03 are MALBAC samples. (ZIP) [file pone.0114520.s011.zip › Figure-S3/CoveragePlot.chr07.png]

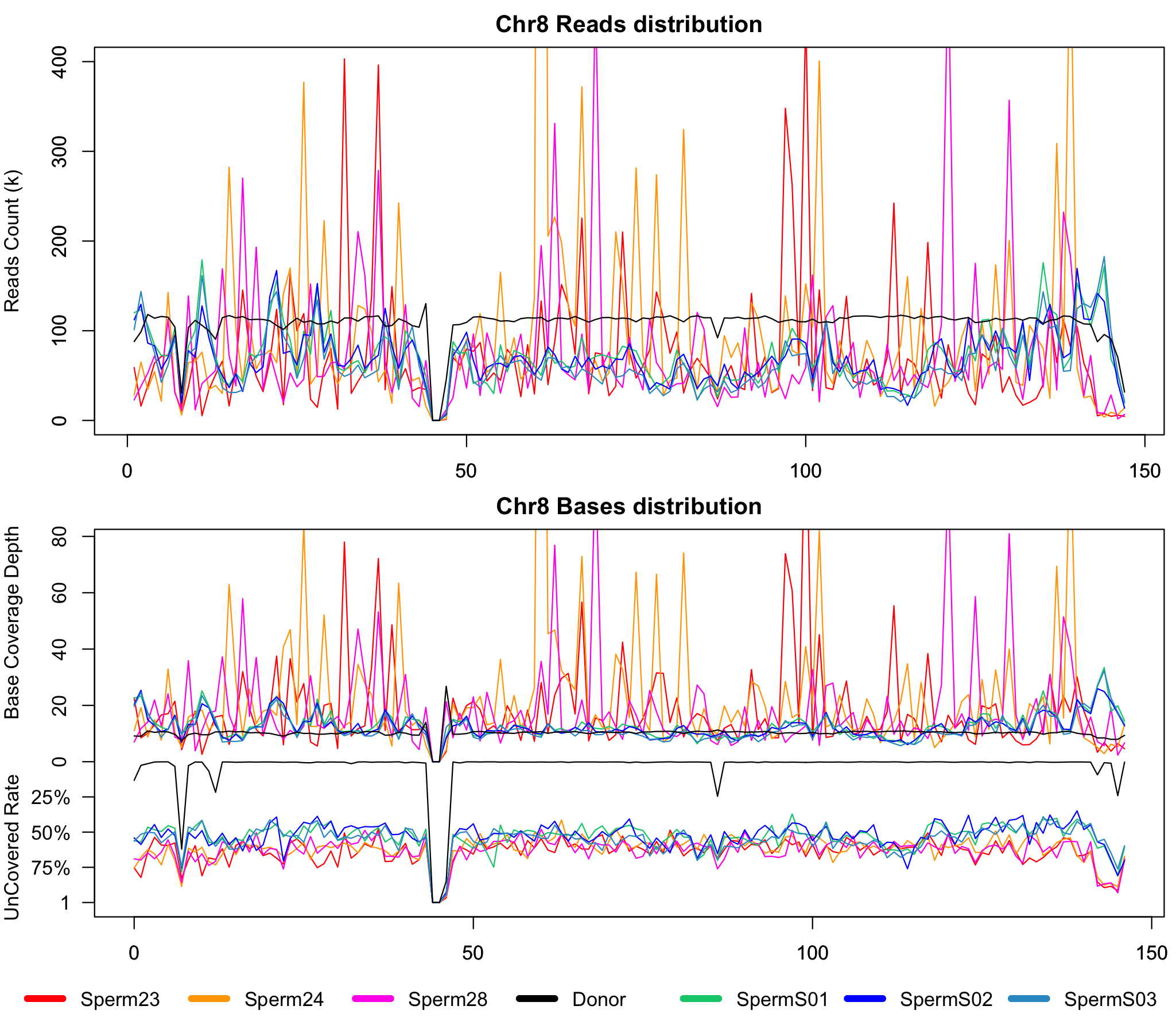

Supplement: S2 File — Genomic coverage on the rest chromosomes. Tilling window size is 1 M. (a) Reads counts in each window. (b) Base coverage depth (upper) and uncovered base rate (lower) in each window. Sperm 23∼28 are MDA samples and Sperm S01∼S03 are MALBAC samples. (ZIP) [file pone.0114520.s011.zip › Figure-S3/CoveragePlot.chr08.png]

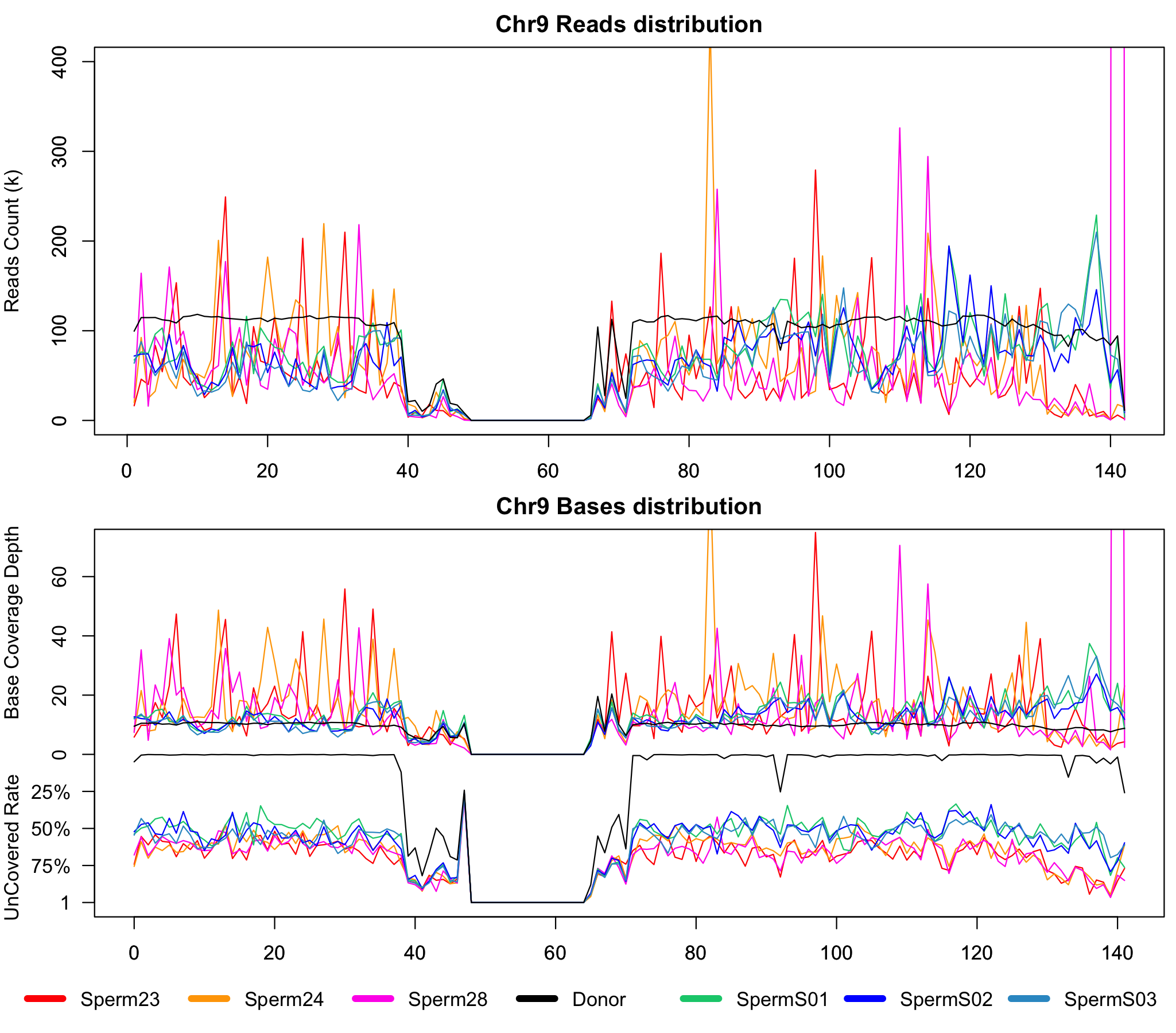

Supplement: S2 File — Genomic coverage on the rest chromosomes. Tilling window size is 1 M. (a) Reads counts in each window. (b) Base coverage depth (upper) and uncovered base rate (lower) in each window. Sperm 23∼28 are MDA samples and Sperm S01∼S03 are MALBAC samples. (ZIP) [file pone.0114520.s011.zip › Figure-S3/CoveragePlot.chr09.png]

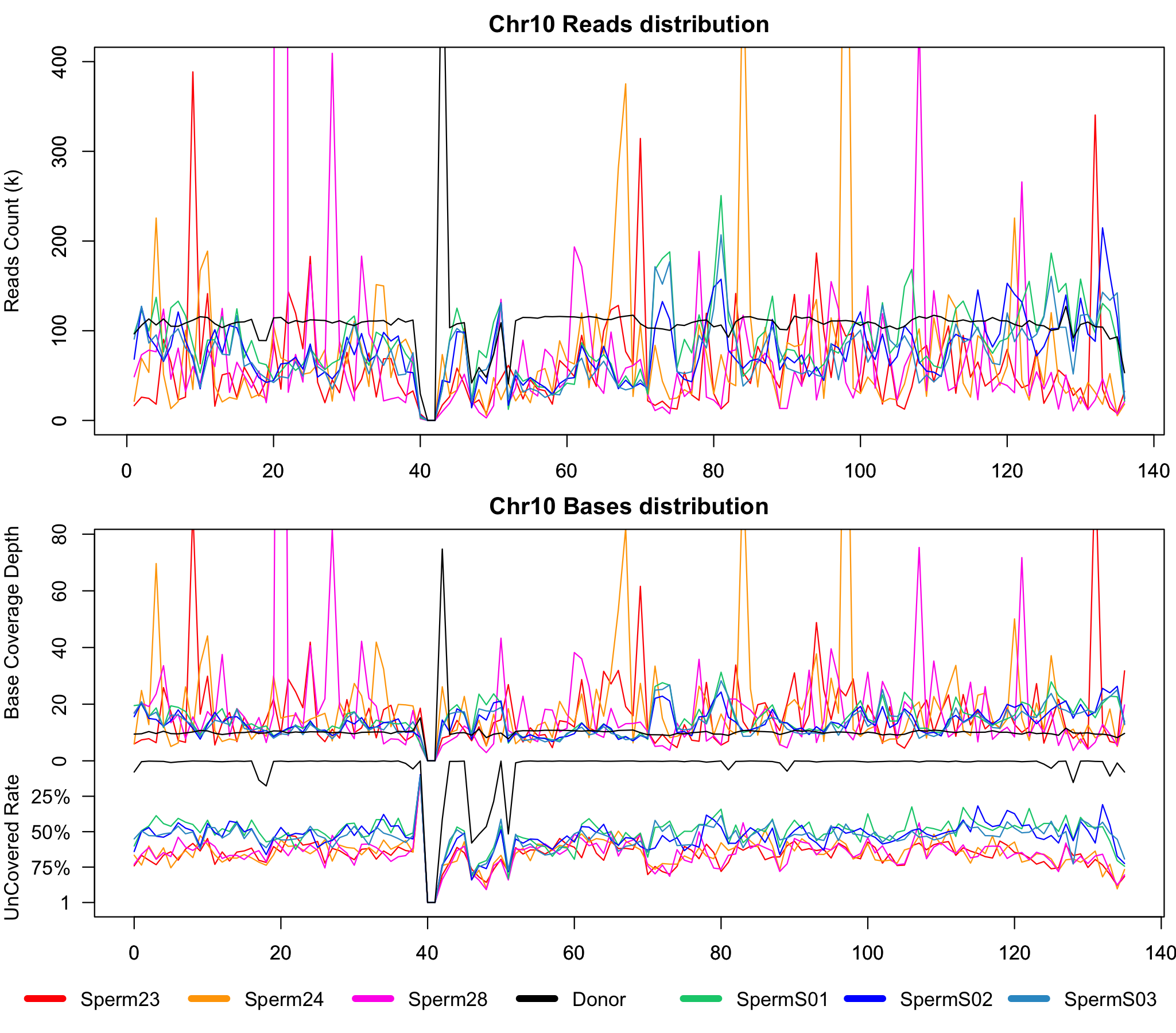

Supplement: S2 File — Genomic coverage on the rest chromosomes. Tilling window size is 1 M. (a) Reads counts in each window. (b) Base coverage depth (upper) and uncovered base rate (lower) in each window. Sperm 23∼28 are MDA samples and Sperm S01∼S03 are MALBAC samples. (ZIP) [file pone.0114520.s011.zip › Figure-S3/CoveragePlot.chr10.png]

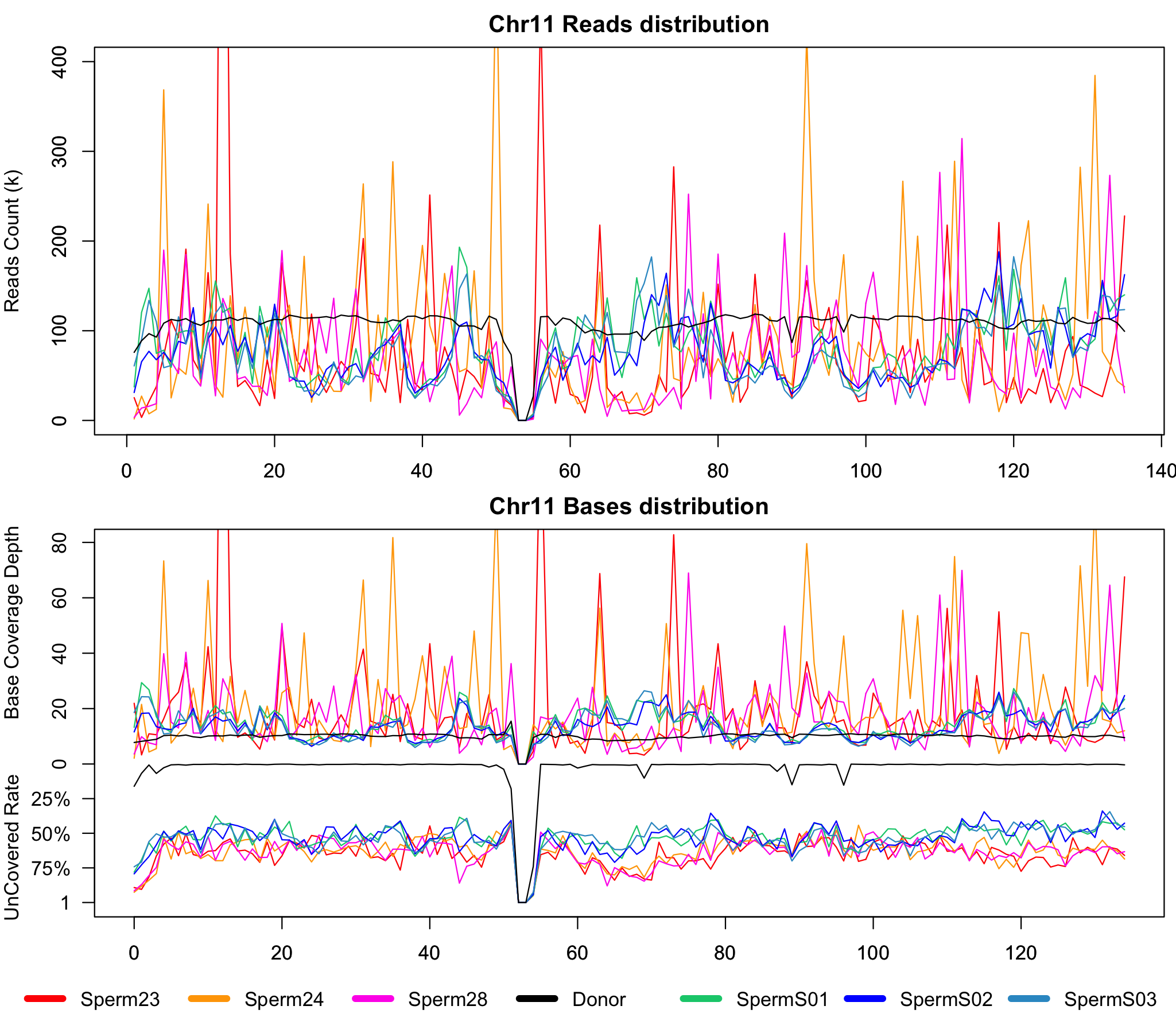

Supplement: S2 File — Genomic coverage on the rest chromosomes. Tilling window size is 1 M. (a) Reads counts in each window. (b) Base coverage depth (upper) and uncovered base rate (lower) in each window. Sperm 23∼28 are MDA samples and Sperm S01∼S03 are MALBAC samples. (ZIP) [file pone.0114520.s011.zip › Figure-S3/CoveragePlot.chr11.png]

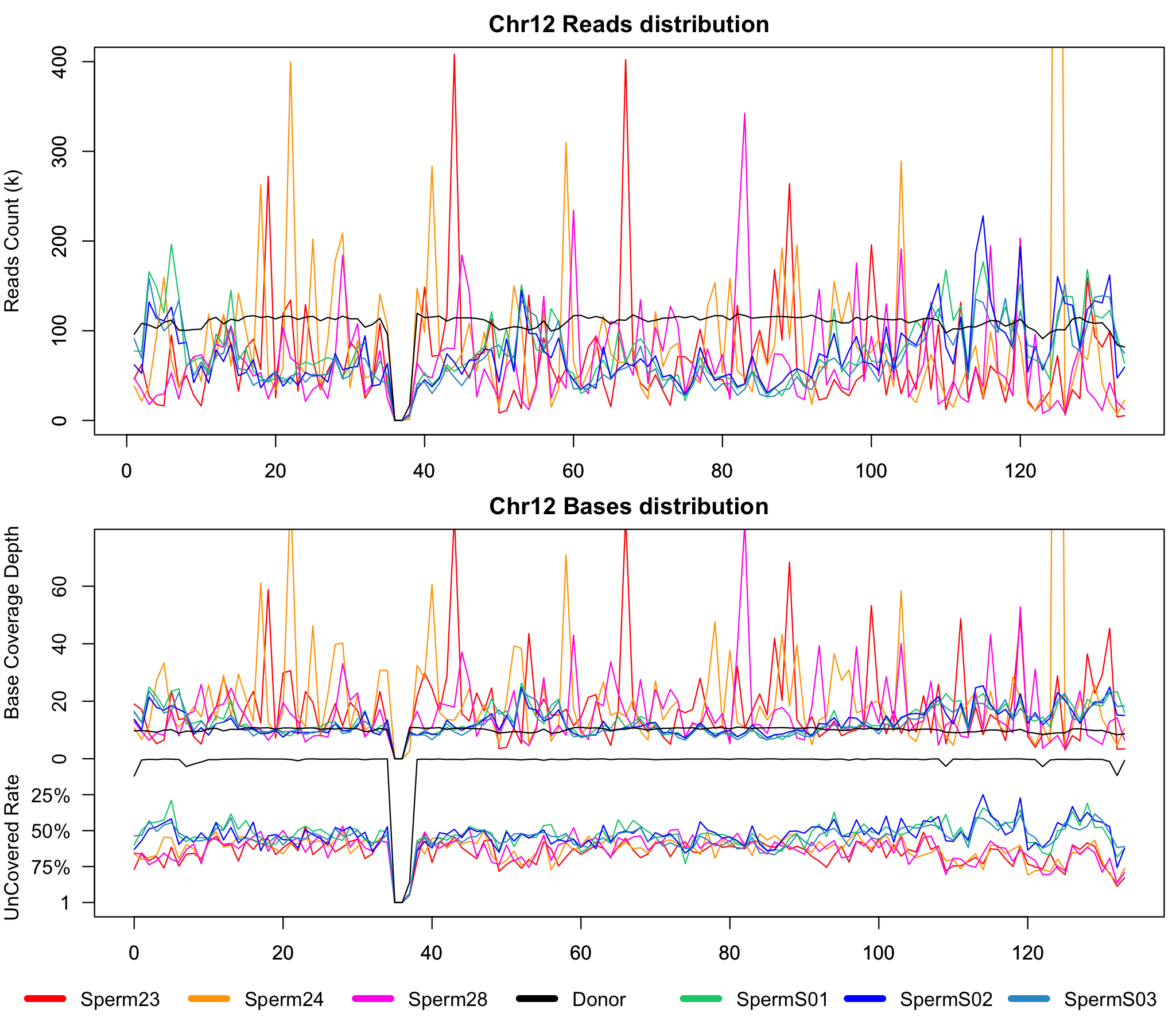

Supplement: S2 File — Genomic coverage on the rest chromosomes. Tilling window size is 1 M. (a) Reads counts in each window. (b) Base coverage depth (upper) and uncovered base rate (lower) in each window. Sperm 23∼28 are MDA samples and Sperm S01∼S03 are MALBAC samples. (ZIP) [file pone.0114520.s011.zip › Figure-S3/CoveragePlot.chr12.png]

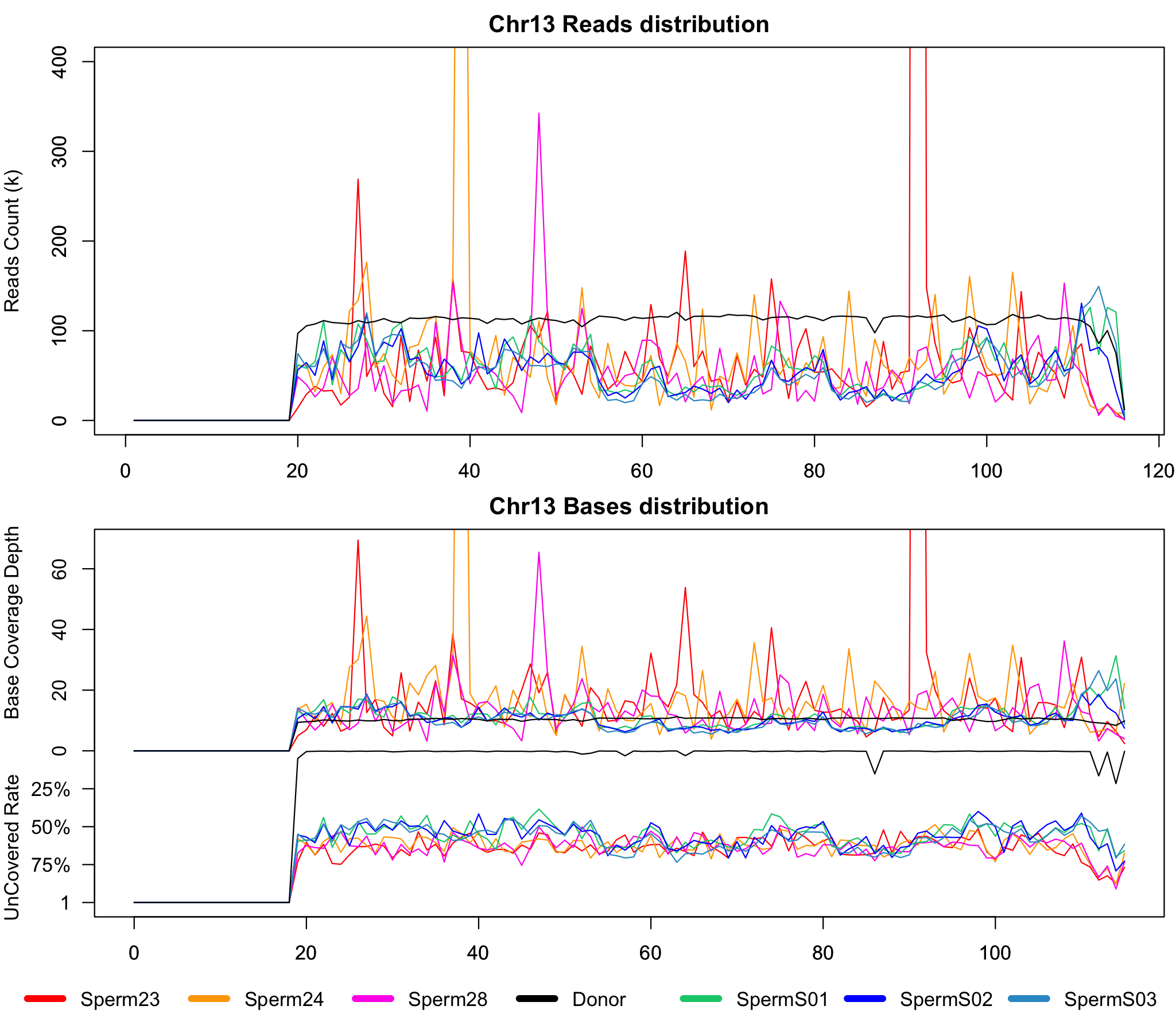

Supplement: S2 File — Genomic coverage on the rest chromosomes. Tilling window size is 1 M. (a) Reads counts in each window. (b) Base coverage depth (upper) and uncovered base rate (lower) in each window. Sperm 23∼28 are MDA samples and Sperm S01∼S03 are MALBAC samples. (ZIP) [file pone.0114520.s011.zip › Figure-S3/CoveragePlot.chr13.png]

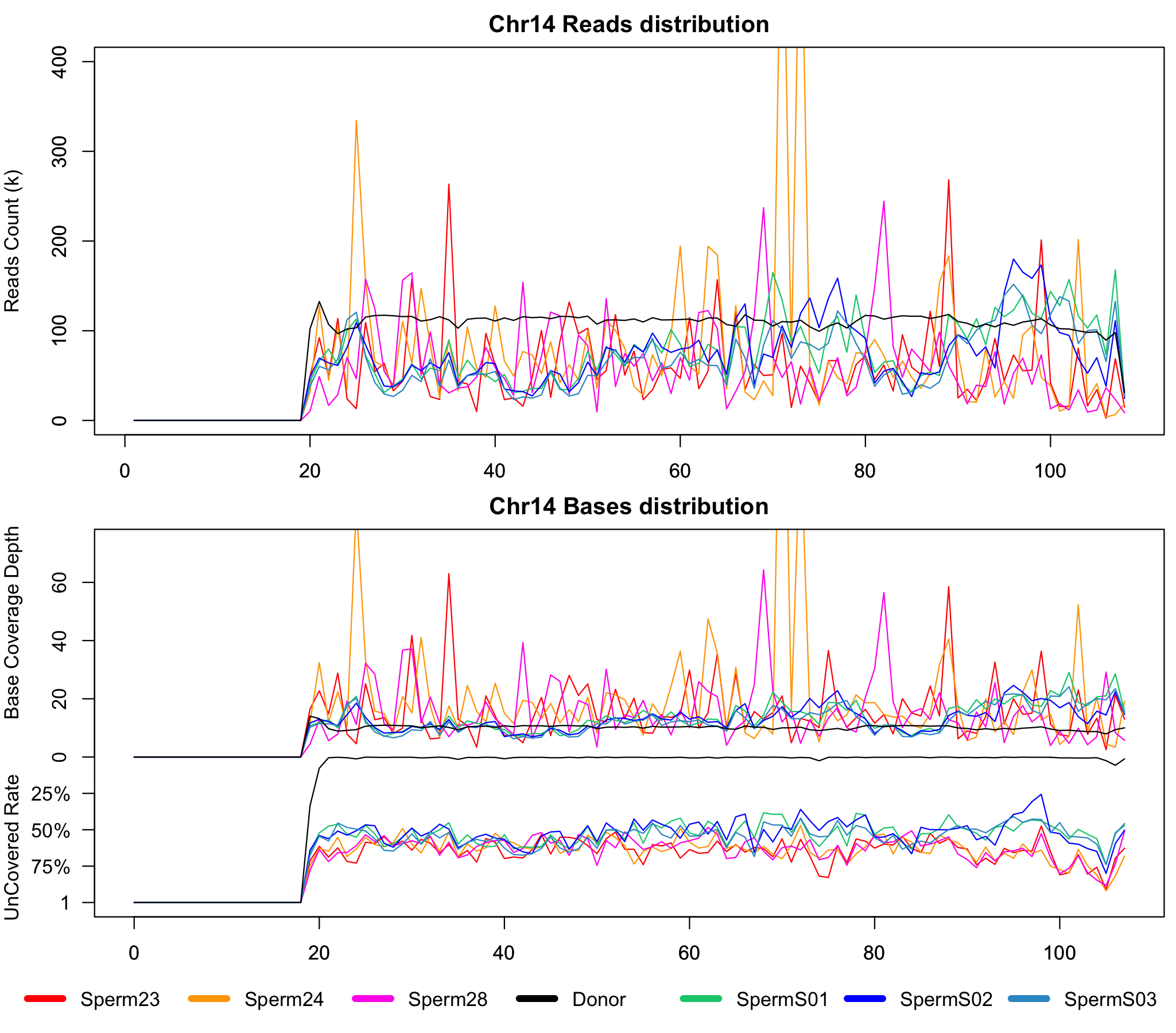

Supplement: S2 File — Genomic coverage on the rest chromosomes. Tilling window size is 1 M. (a) Reads counts in each window. (b) Base coverage depth (upper) and uncovered base rate (lower) in each window. Sperm 23∼28 are MDA samples and Sperm S01∼S03 are MALBAC samples. (ZIP) [file pone.0114520.s011.zip › Figure-S3/CoveragePlot.chr14.png]

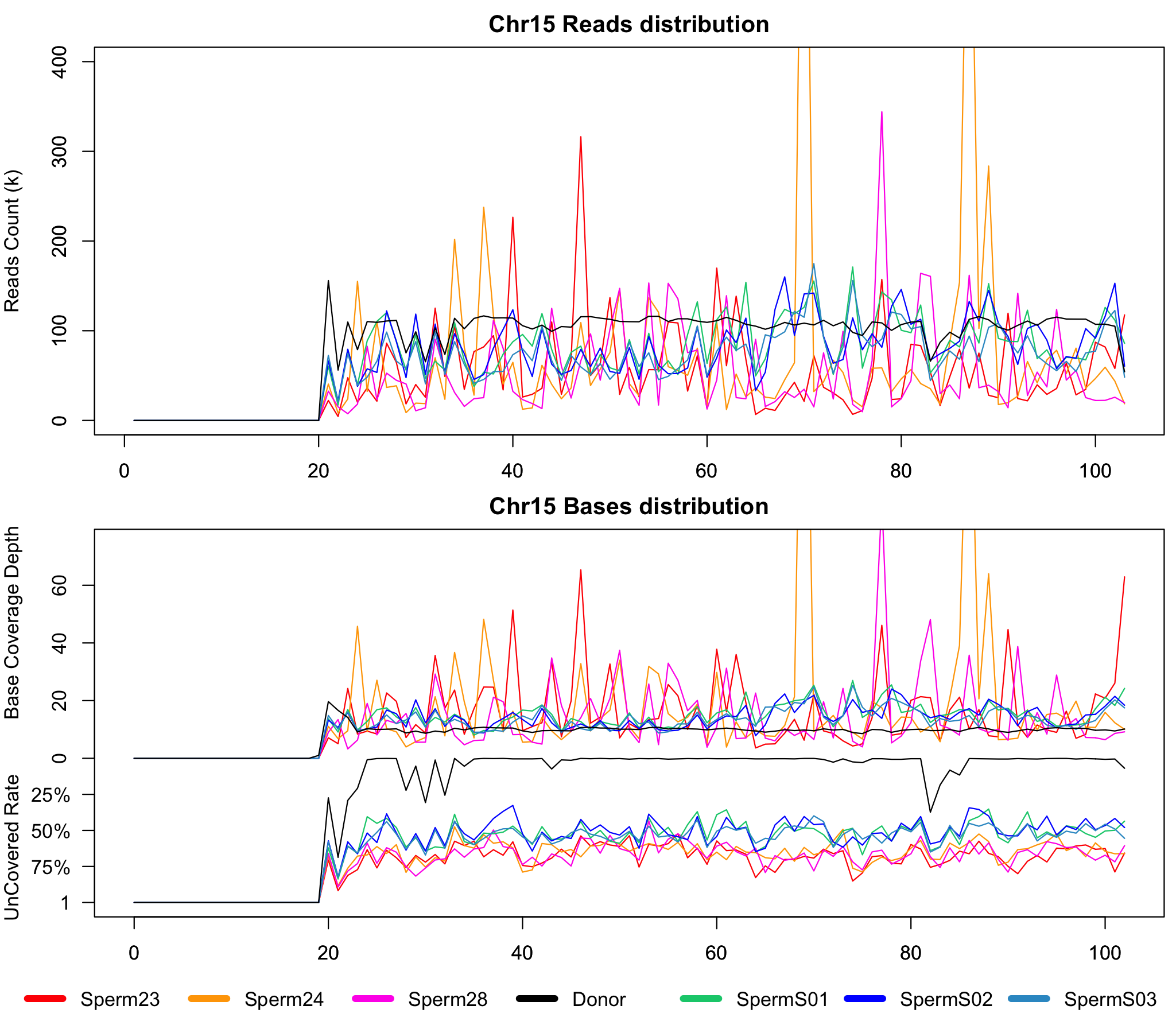

Supplement: S2 File — Genomic coverage on the rest chromosomes. Tilling window size is 1 M. (a) Reads counts in each window. (b) Base coverage depth (upper) and uncovered base rate (lower) in each window. Sperm 23∼28 are MDA samples and Sperm S01∼S03 are MALBAC samples. (ZIP) [file pone.0114520.s011.zip › Figure-S3/CoveragePlot.chr15.png]

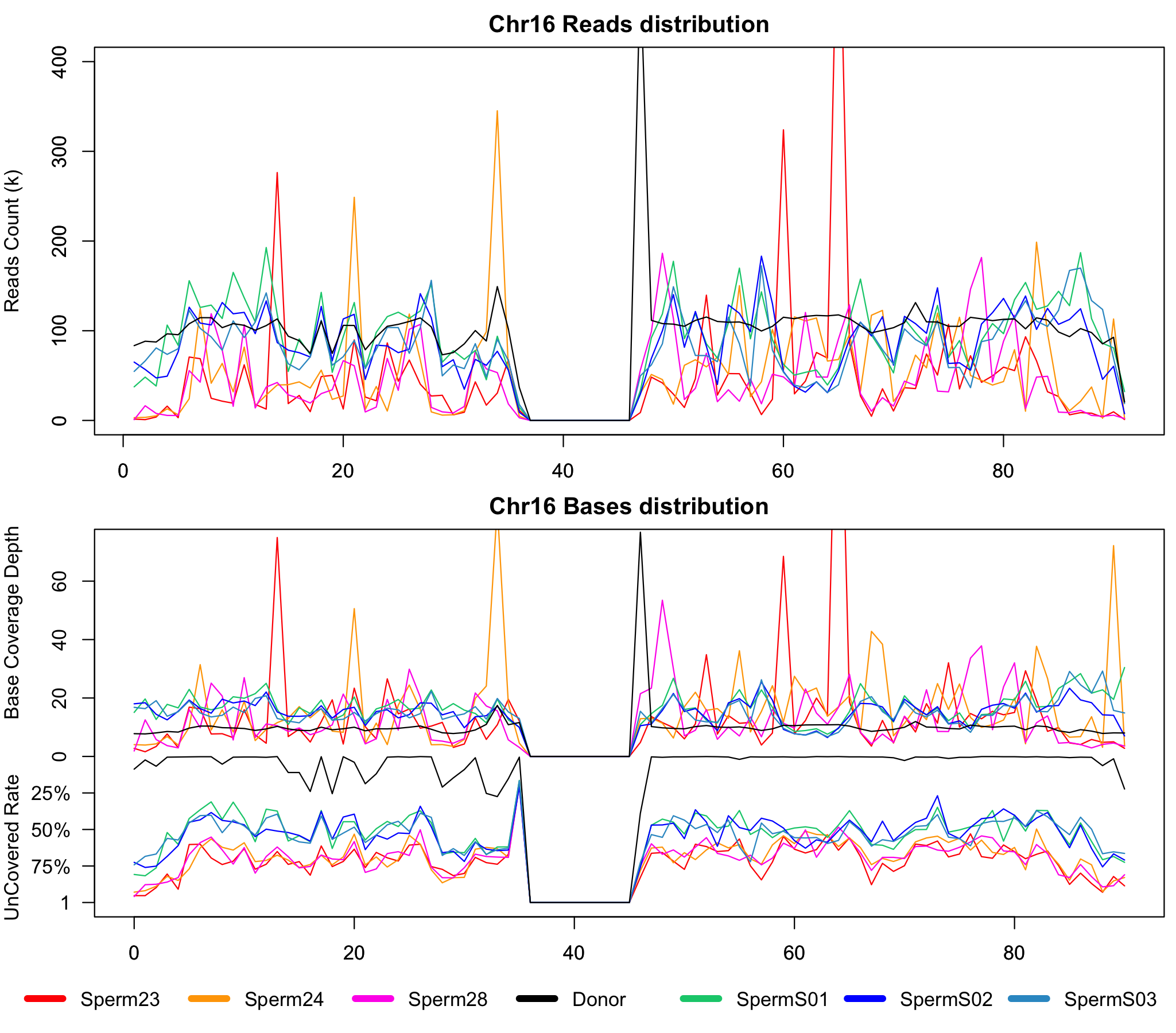

Supplement: S2 File — Genomic coverage on the rest chromosomes. Tilling window size is 1 M. (a) Reads counts in each window. (b) Base coverage depth (upper) and uncovered base rate (lower) in each window. Sperm 23∼28 are MDA samples and Sperm S01∼S03 are MALBAC samples. (ZIP) [file pone.0114520.s011.zip › Figure-S3/CoveragePlot.chr16.png]

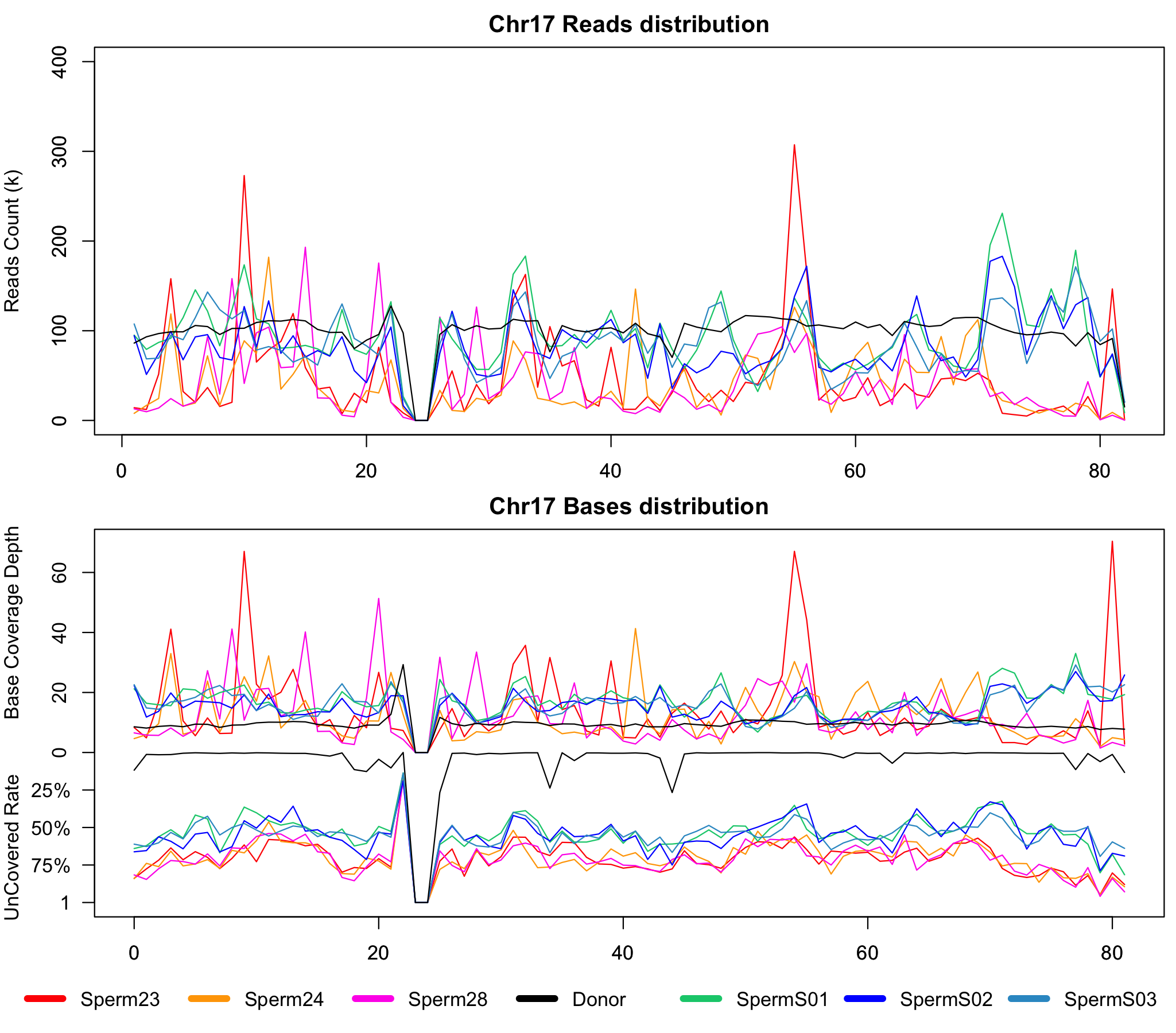

Supplement: S2 File — Genomic coverage on the rest chromosomes. Tilling window size is 1 M. (a) Reads counts in each window. (b) Base coverage depth (upper) and uncovered base rate (lower) in each window. Sperm 23∼28 are MDA samples and Sperm S01∼S03 are MALBAC samples. (ZIP) [file pone.0114520.s011.zip › Figure-S3/CoveragePlot.chr17.png]

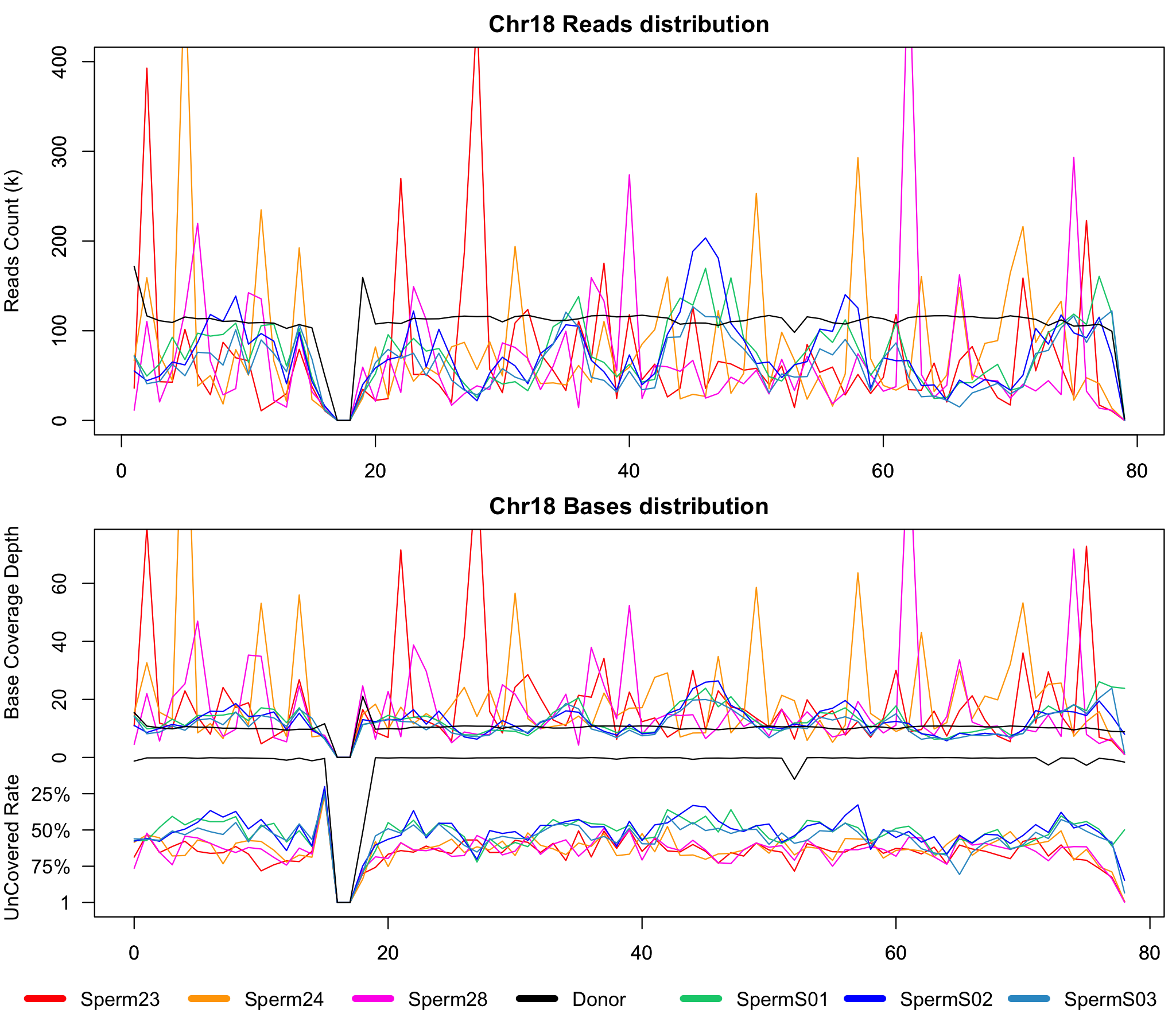

Supplement: S2 File — Genomic coverage on the rest chromosomes. Tilling window size is 1 M. (a) Reads counts in each window. (b) Base coverage depth (upper) and uncovered base rate (lower) in each window. Sperm 23∼28 are MDA samples and Sperm S01∼S03 are MALBAC samples. (ZIP) [file pone.0114520.s011.zip › Figure-S3/CoveragePlot.chr18.png]

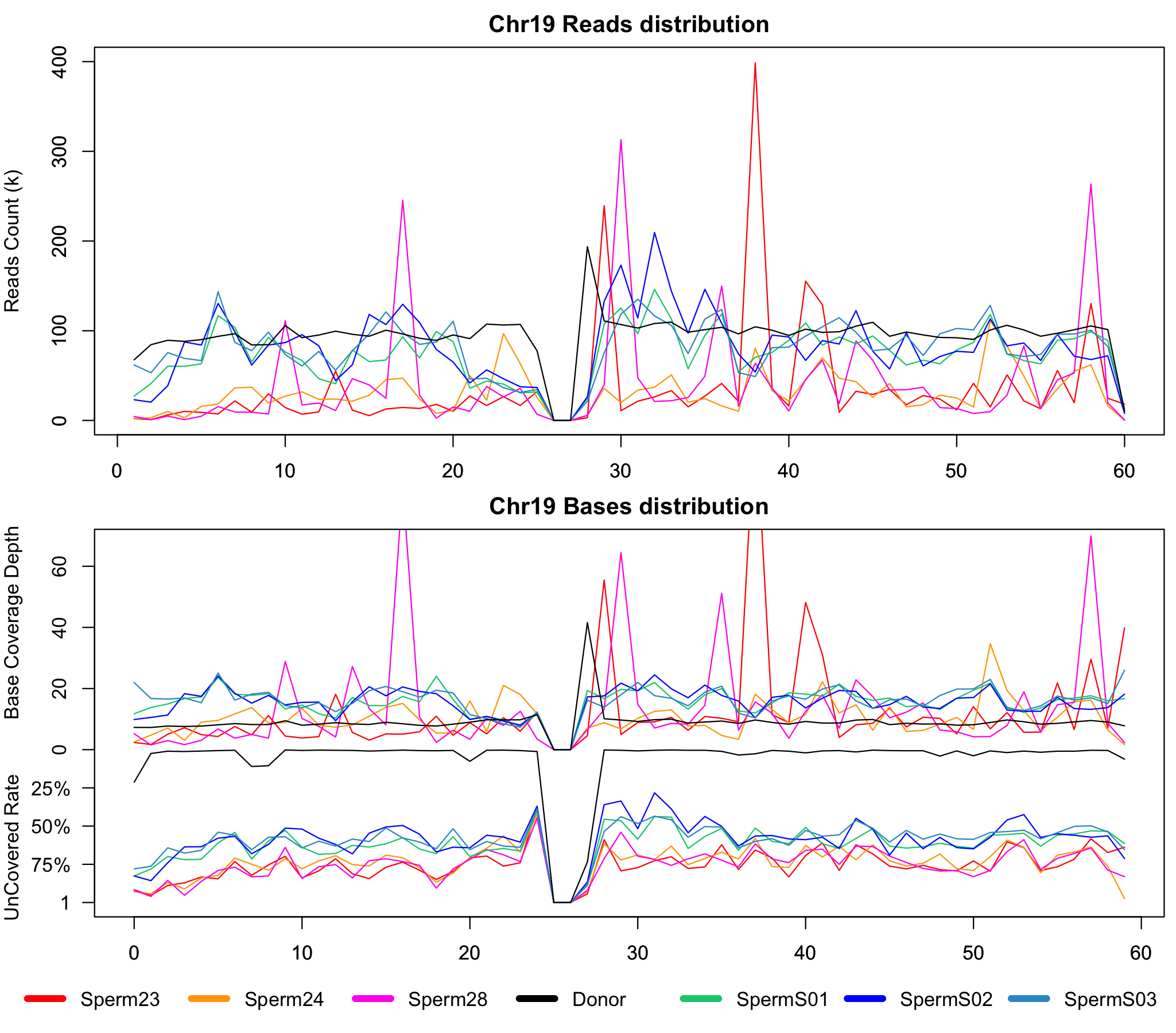

Supplement: S2 File — Genomic coverage on the rest chromosomes. Tilling window size is 1 M. (a) Reads counts in each window. (b) Base coverage depth (upper) and uncovered base rate (lower) in each window. Sperm 23∼28 are MDA samples and Sperm S01∼S03 are MALBAC samples. (ZIP) [file pone.0114520.s011.zip › Figure-S3/CoveragePlot.chr19.png]

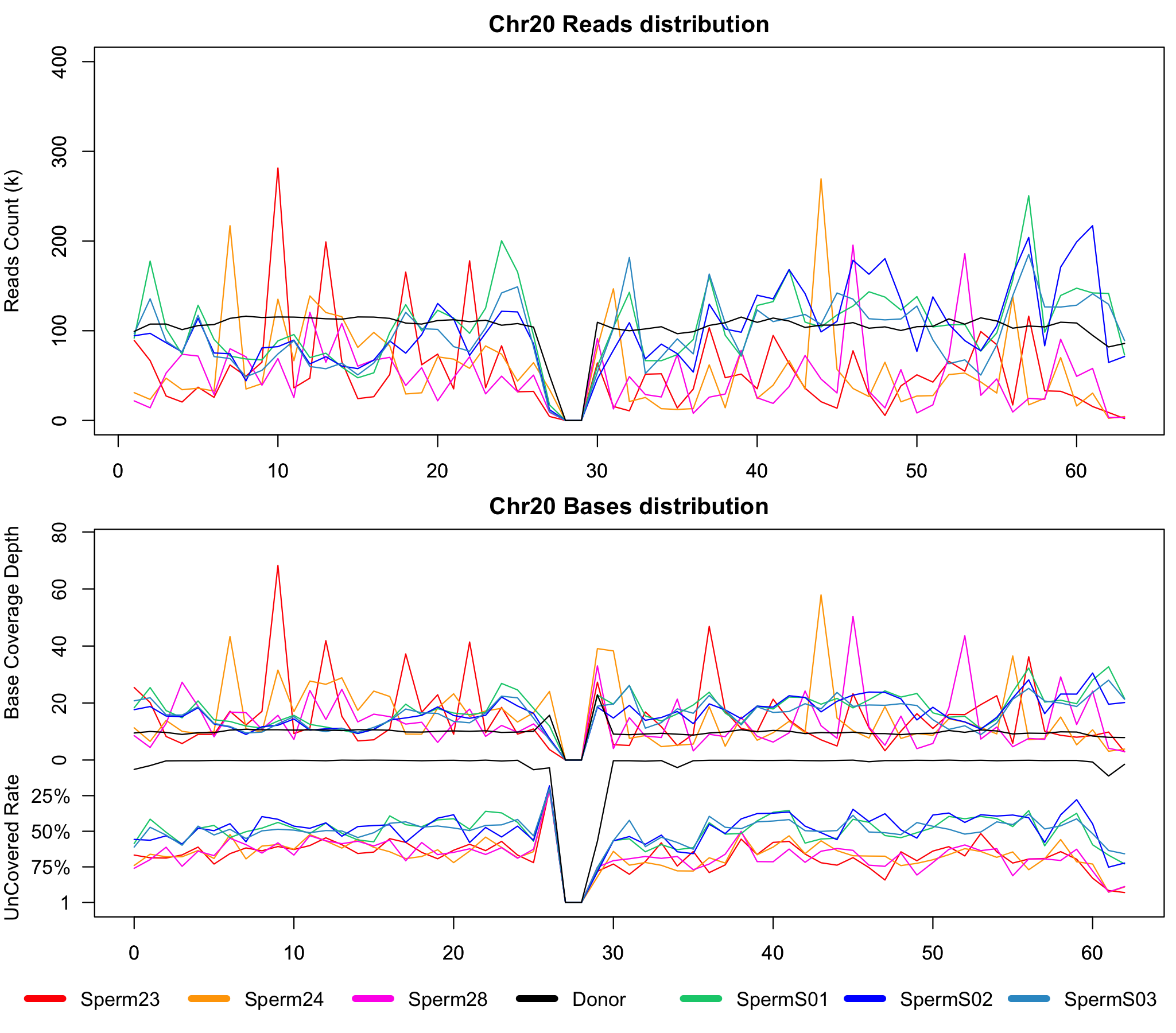

Supplement: S2 File — Genomic coverage on the rest chromosomes. Tilling window size is 1 M. (a) Reads counts in each window. (b) Base coverage depth (upper) and uncovered base rate (lower) in each window. Sperm 23∼28 are MDA samples and Sperm S01∼S03 are MALBAC samples. (ZIP) [file pone.0114520.s011.zip › Figure-S3/CoveragePlot.chr20.png]

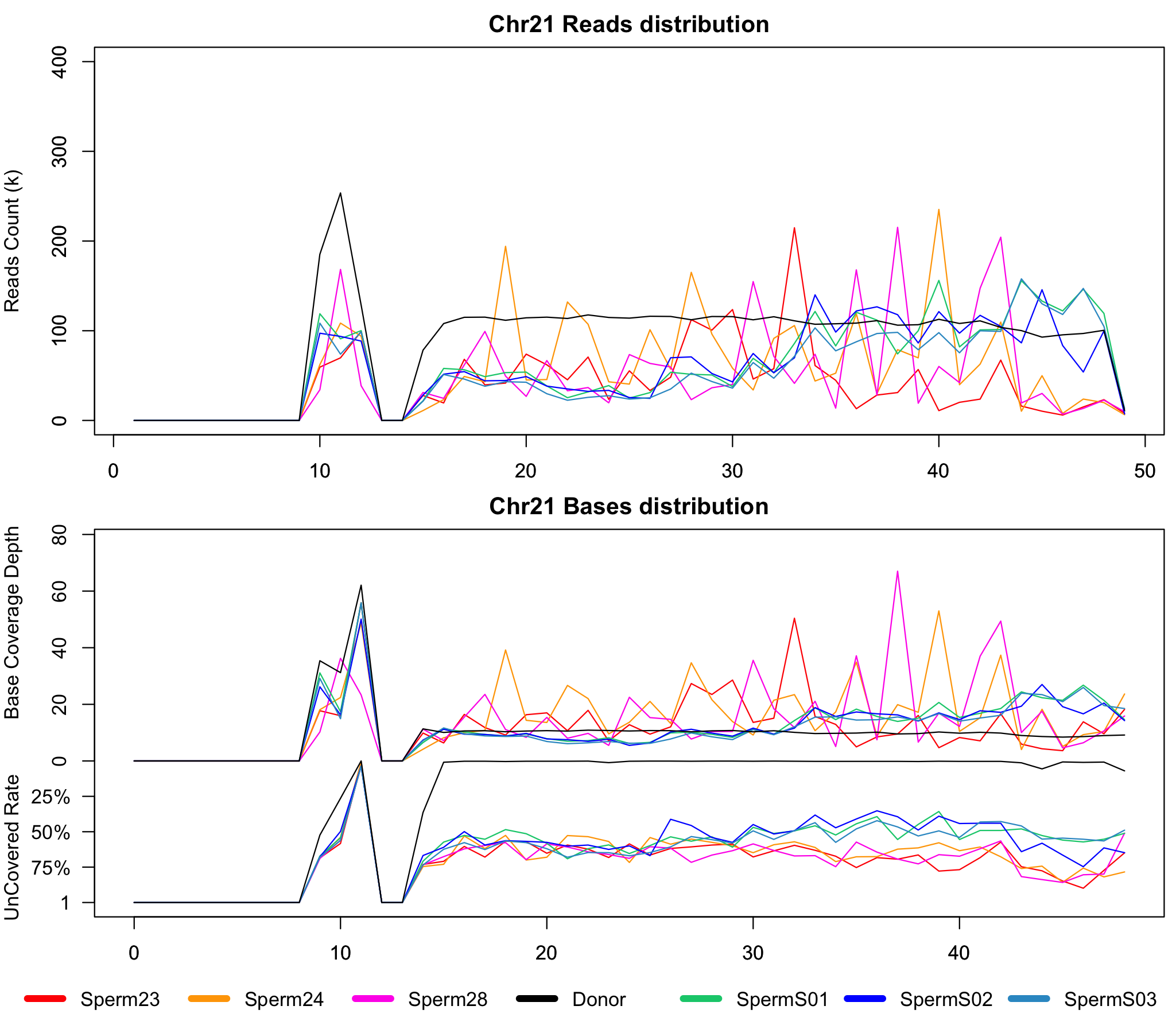

Supplement: S2 File — Genomic coverage on the rest chromosomes. Tilling window size is 1 M. (a) Reads counts in each window. (b) Base coverage depth (upper) and uncovered base rate (lower) in each window. Sperm 23∼28 are MDA samples and Sperm S01∼S03 are MALBAC samples. (ZIP) [file pone.0114520.s011.zip › Figure-S3/CoveragePlot.chr21.png]

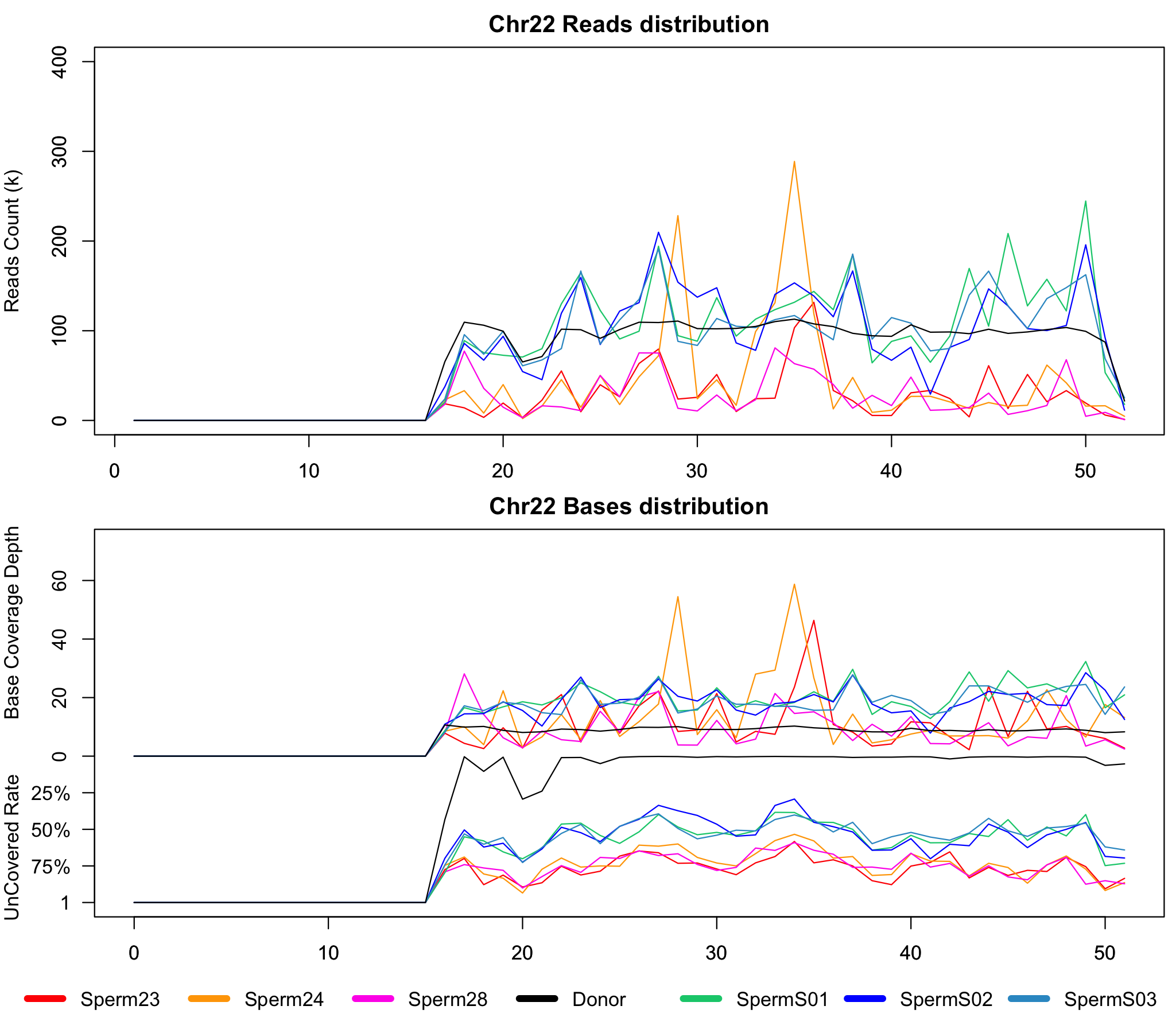

Supplement: S2 File — Genomic coverage on the rest chromosomes. Tilling window size is 1 M. (a) Reads counts in each window. (b) Base coverage depth (upper) and uncovered base rate (lower) in each window. Sperm 23∼28 are MDA samples and Sperm S01∼S03 are MALBAC samples. (ZIP) [file pone.0114520.s011.zip › Figure-S3/CoveragePlot.chr22.png]
