# Supplementary material for: Comparison of Multiple Displacement Amplification (MDA) and Multiple Annealing and Looping-Based Amplification Cycles (MALBAC) in Single-Cell Sequencing
Source: PLoS One. 2014 Dec 8;9(12):e114520. doi: 10.1371/journal.pone.0114520 (PMC4259343; doi:10.1371/journal.pone.0114520)
Supplement: S1 Table — Sample list with accession numbers. (DOCX) [file pone.0114520.s003.docx]

## Table S1, Sample list with accession numbers.

| Type | Sample Name | SRA accession | Raw Bases |
| --- | --- | --- | --- |
| **MDA** | Sperm23 | SRR504410, SRR504411 | 27,934,970,382 |
| **MDA** | Sperm24 | SRR504412, SRR504413 | 27,422,893,872 |
| **MDA** | Sperm28 | SRR504421, SRR504422 | 27,062,454,276 |
| **MALBAC** | SpermS01 | SRR618565 | 26,848,746,200 |
| **MALBAC** | SpermS02 | SRR618566 | 26,734,894,000 |
| **MALBAC** | SpermS03 | SRR618567 | 23,988,296,200 |
| **WGS** | Donor | SRR618666 | 46,257,111,600 |
