# Supplementary material for: Comparison of Multiple Displacement Amplification (MDA) and Multiple Annealing and Looping-Based Amplification Cycles (MALBAC) in Single-Cell Sequencing
Source: PLoS One. 2014 Dec 8;9(12):e114520. doi: 10.1371/journal.pone.0114520 (PMC4259343; doi:10.1371/journal.pone.0114520)
Supplement: S2 Table — Goodness-of-fit test to match K-mer distribution to Possion probability mass function of the theoretic lambda and compare MDA & MALBAC K-mer distribution to the blood K-mer distribution with Kolmogorov-Smirnov test. (DOCX) [file pone.0114520.s004.docx]

## Table S2. Goodness-of-fit test to compare K-mer distribution to Possion probability mass function of the theoretic lambda and compare MDA & MALBAC K-mer distribution to the blood K-mer distribution with Kolmogorov-Smirnov test.

|  | Theoretic 𝜆* | 𝜒^2^ | *P*-value | D to blood | *P*-value |
| --- | --- | --- | --- | --- | --- |
| MDA 23 | 3.26 | 116 | 1.54E-18 | 0.47 | 0.02 |
| MDA 24 | 3.28 | 113 | 5.12E-18 | 0.47 | 0.02 |
| MDA 28 | 3.31 | 981 | 3.86E-15 | 0.47 | 0.02 |
| Donor | 3.28 | 1.00 | 1.00E+00 | - | - |
| MALBAC 01 | 3.30 | 99.8 | 1.78E-15 | 0.53 | 0.009 |
| MALBAC 02 | 3.28 | 104 | 2.22E-16 | 0.53 | 0.009 |
| MALBAC 03 | 2.94 | 313 | 3.71E-59 | 0.53 | 0.009 |

We only choose count [2,15] for the calculation of Pearson 𝜒^2^ test, thus df = 13. Theoretic 𝜆 is calculated from sequencing depth of reads, which are used for k-mer counting.
