# Supplementary material for: Comparison of Multiple Displacement Amplification (MDA) and Multiple Annealing and Looping-Based Amplification Cycles (MALBAC) in Single-Cell Sequencing
Source: PLoS One. 2014 Dec 8;9(12):e114520. doi: 10.1371/journal.pone.0114520 (PMC4259343; doi:10.1371/journal.pone.0114520)
Supplement: S3 Table — The ratio of single-cell sequencing and mapping in diferent samples. (DOCX) [file pone.0114520.s005.docx]

## Table S3. The ratio of single-cell sequencing and mapping in diferent samples.

| Sample | Raw Bases (G bp) | Reads mapped rate | Bases mapped rate * | Mapped reads unique rate | Unique mapped Coverage Ratio ** | Unique average mapped reads length ** |
| --- | --- | --- | --- | --- | --- | --- |
| **MDA 23** | 27.93 | 91.57% | 87.94% | 96.95% | 34.73% | 95.07 |
| **MDA 24** | 27.42 | 87.98% | 84.75% | 96.55% | 34.94% | 95.36 |
| **MDA 28** | 27.06 | 91.46% | 87.57% | 97.10% | 35.98% | 94.80 |
| **Donor** | 46.26 | 89.73% | 81.72% | 95.59% | 98.00% | 91.41 |
| **MALBAC 01** | 26.85 | 94.79% | 78.88% | 96.47% | 45.20% | 83.38 |
| **MALBAC 02** | 26.73 | 95.00% | 79.27% | 96.71% | 47.02% | 83.62 |
| **MALBAC 03** | 23.99 | 94.83% | 79.89% | 96.57% | 43.31% | 84.39 |

Star marks show significant level between MDA group and MALBAC group, using 2-tailed unequal variances *t*-test.
