# Supplementary material for: Comparison of Multiple Displacement Amplification (MDA) and Multiple Annealing and Looping-Based Amplification Cycles (MALBAC) in Single-Cell Sequencing
Source: PLoS One. 2014 Dec 8;9(12):e114520. doi: 10.1371/journal.pone.0114520 (PMC4259343; doi:10.1371/journal.pone.0114520)
Supplement: S4 Table — Coefficient of variation of coverage in 1Mb windows on autosomes. (DOCX) [file pone.0114520.s006.docx]

## Table S4. Coefficient of variation of coverage in 1Mb windows on autosomes.

|  | Reads Count | Base Coverage Depth | Uncovered Rate |
| --- | --- | --- | --- |
| MDA 23 | 8.1695 | 1.2672 | 0.1608 |
| MDA 24 | 1.5307 | 1.2495 | 0.1711 |
| MDA 28 | 4.1640 | 1.2318 | 0.1693 |
| Donor | 0.5141 | 0.4258 | 3.4266 |
| MALBAC 01 | 0.5325 | 0.4540 | 0.2427 |
| MALBAC 02 | 0.5150 | 0.4264 | 0.2415 |
| MALBAC 03 | 0.5545 | 0.4681 | 0.2177 |
| *t*-test for sperms | 0.1016 | 9.468E-07** | 1.565E-03** |

Assuming different variance in 2-tail t-test.
