# Supplementary material for: Comparison of Multiple Displacement Amplification (MDA) and Multiple Annealing and Looping-Based Amplification Cycles (MALBAC) in Single-Cell Sequencing
Source: PLoS One. 2014 Dec 8;9(12):e114520. doi: 10.1371/journal.pone.0114520 (PMC4259343; doi:10.1371/journal.pone.0114520)
Supplement: S5 Table — Pairwise Kendall's τ coefficient test of base coverage of different samples on autosomes. (DOCX) [file pone.0114520.s007.docx]

## Table S5. Pairwise Kendall's τ coefficient test of bases coverage of different samples on autosomes.

|  | MDA 23 | MDA 24 | MDA 28 | Donor | MALBAC 01 | MALBAC 02 | MALBAC 03 |
| --- | --- | --- | --- | --- | --- | --- | --- |
| MDA 23 | 1 | 0.2316 | 0.2260 | 0.2889 | -0.0017** | 0.0144** | -0.0219** |
| MDA 24 |  | 1 | 0.2287 | 0.2852 | 0.0067** | 0.0267* | -0.0144** |
| MDA 28 |  |  | 1 | 0.2857 | 0.0058** | 0.0297* | -0.0143** |
| Donor |  |  |  | 1 | -0.1649 | -0.1287 | -0.1989 |
| MALBAC 01 |  |  |  |  | 1 | 0.7601 | 0.7997 |
| MALBAC 02 |  |  |  |  |  | 1 | 0.7669 |
| MALBAC 03 |  |  |  |  |  |  | 1 |

* and ** means p more than 1% and 5%. Alternative hypothesis: true tau is not equal to 0.
