# Supplementary material for: Comparison of Multiple Displacement Amplification (MDA) and Multiple Annealing and Looping-Based Amplification Cycles (MALBAC) in Single-Cell Sequencing
Source: PLoS One. 2014 Dec 8;9(12):e114520. doi: 10.1371/journal.pone.0114520 (PMC4259343; doi:10.1371/journal.pone.0114520)
Supplement: S6 Table — Statistics of SNP calling in different samples. (DOCX) [file pone.0114520.s008.docx]

## Table S6. Statistics of SNP calling in different samples.

| **Sample** | **Samtools+Bcftools** | | | | **Soapsnp** | | | |
| --- | --- | --- | --- | --- | --- | --- | --- | --- |
|  | **Hom** | **Het** | **Sum** | **Het %** | **Hom** | **Het** | **Sum** | **Het %** |
| **MDA 23** | 296,565 | 27,122 | 323,687 | 8.38% | 267,851 | 15,114 | 282,965 | 5.34% |
| **MDA 24** | 324,417 | 25,783 | 350,200 | 7.36% | 287,271 | 16,600 | 303,871 | 5.46% |
| **MDA 28** | 298,708 | 32,105 | 330,813 | 9.70% | 272,782 | 17,663 | 290,445 | 6.08% |
| **MALBAC 01** | 407,263 | 432,465 | 839,728 | 51.50% | 459,457 | 432,957 | 892,414 | 48.52% |
| **MALBAC 02** | 381,178 | 410,371 | 791,549 | 51.84% | 465,290 | 467,852 | 933,142 | 50.14% |
| **MALBAC 03** | 404,960 | 379,122 | 784,082 | 48.35% | 441,589 | 370,343 | 811,932 | 45.61% |
