# Supplementary material for: Comparison of Multiple Displacement Amplification (MDA) and Multiple Annealing and Looping-Based Amplification Cycles (MALBAC) in Single-Cell Sequencing
Source: PLoS One. 2014 Dec 8;9(12):e114520. doi: 10.1371/journal.pone.0114520 (PMC4259343; doi:10.1371/journal.pone.0114520)
Supplement: S7 Table — Distribution of allele types in different samples. (DOCX) [file pone.0114520.s009.docx]

## Table S7. Distribution of allele types in different samples.

|  | **S01** | **S02** | **S03** | **S23** | **S24** | **S28** |
| --- | --- | --- | --- | --- | --- | --- |
| **A>C** | 37078 | 39744 | 33552 | 10321 | 11019 | 10544 |
| **A>G** | 119041 | 124001 | 109368 | 40027 | 43104 | 41679 |
| **A>T** | 61127 | 66412 | 52627 | 8771 | 9421 | 9228 |
| **C>A** | 48242 | 49770 | 44580 | 10506 | 11230 | 10896 |
| **C>G** | 33860 | 34969 | 31266 | 10244 | 10882 | 10540 |
| **C>T** | 126813 | 132187 | 117444 | 41241 | 43962 | 42270 |
| **G>A** | 127157 | 131087 | 117723 | 41633 | 44416 | 42579 |
| **G>C** | 34934 | 36532 | 32197 | 10244 | 11147 | 10691 |
| **G>T** | 48135 | 50407 | 44082 | 10360 | 11467 | 10967 |
| **T>A** | 61113 | 66001 | 52966 | 8954 | 9357 | 9329 |
| **T>C** | 120700 | 126531 | 111201 | 40610 | 43069 | 41554 |
| **T>G** | 35869 | 37968 | 32061 | 10349 | 11260 | 11116 |
| **SUM** | 854069 | 895609 | 779067 | 243260 | 260334 | 251393 |
